# Non-parametric frailty Cox models for hierarchical time-to-event data

**DOI:** 10.1093/biostatistics/kxy071

**Published:** 2018-12-26

**Authors:** Francesca Gasperoni, Francesca Ieva, Anna Maria Paganoni, Christopher H Jackson, Linda Sharples

**Affiliations:** 1 MOX - Modelling and Scientific Computing, Department of Mathematics Politecnico di Milano, Piazza Leonardo Da Vinci 32, Milano 20123, Italy; 2 MRC Biostatistics Unit, Cambridge Institute of Public Health, Forvie Site, Robinson Way, Cambridge Biomedical Campus, Cambridge CB2 0SR, UK; 3 Department of Medical Statistics, London School of Hygiene & Tropical Medicine, Keppel Street, London WC1E 7HT, UK

**Keywords:** Discrete frailty, Expectation–Maximization algorithm, Finite mixture model, Multilevel survival data, Time-to-event data

## Abstract

We propose a novel model for hierarchical time-to-event data, for example, healthcare data in which patients are grouped by their healthcare provider. The most common model for this kind of data is the Cox proportional hazard model, with frailties that are common to patients in the same group and given a parametric distribution. We relax the parametric frailty assumption in this class of models by using a non-parametric discrete distribution. This improves the flexibility of the model by allowing very general frailty distributions and enables the data to be clustered into groups of healthcare providers with a similar frailty. A tailored Expectation–Maximization algorithm is proposed for estimating the model parameters, methods of model selection are compared, and the code is assessed in simulation studies. This model is particularly useful for administrative data in which there are a limited number of covariates available to explain the heterogeneity associated with the risk of the event. We apply the model to a clinical administrative database recording times to hospital readmission, and related covariates, for patients previously admitted once to hospital for heart failure, and we explore latent clustering structures among healthcare providers.

## 1. Introduction

Time-to-event methods are used extensively in medical statistics, with the Cox proportional hazards model providing both flexibility and tractability, and requiring only that the proportional hazards assumption is valid (Cox, 1972). Extensions to this model to allow for the common situation of clustering of individuals (or shared frailty), for example due to repeated assessments of patients within the same healthcare provider, have been developed (Hougaard, 2000). Published examples include survival of patients grouped in hospitals (Austin, 2017) and time to udder infection in cows, with the four mammary glands making up the udder grouped as individuals (Geerdens *and others*, 2013). These examples rely on a parametric form for the frailty distribution, such as the Gamma or log-Normal. However, a non-parametric alternative is desirable, due to potential misspecification of the parametric form and as a method for detecting clusters of groups with similar frailties, which is the goal of this work.

This work is motivated by the analysis of times to the second admission to a healthcare provider (such as hospital, research center, or nursing home) for heart failure (HF) patients in the Lombardia region of Italy. Specifically, we wanted to undertake an exploratory analysis for detecting and investigating clusters of healthcare providers, adjusting for patient-specific covariates. To the best of our knowledge, most literature regarding profiling of healthcare providers does not exploit time-to-event data but is typically based on multilevel logistic regression of binary outcomes on patient-level and structure-level covariates (Grieco *and others*, 2011).

A review of models for multilevel time-to-event data was done by Duchateau and Janssen (2007) and Hougaard (2000), including both subject-specific and group-specific (shared) parametric frailty models. The most common distributions for the frailty term are Gamma and Log-Normal, probably due their analytical tractability and software availability [see package $\texttt{coxph in survival}$ by Therneau and Grambsch (2000) and Therneau (2014)]. Positive stable and power variance distributions have also become accessible through the package $\texttt{frailtyEM}$ (Balan and Putter, 2017). All the mentioned packages are developed in R (R Development Core Team, 2016).

Only a few publications have considered discrete frailties, and we are not aware of any general software for fitting discrete frailty models. Several authors describe discrete frailty models in a frequentist framework, though typically with a parametric baseline survival function or subject-specific frailties. Caroni *and others* (2010) used a Weibull baseline and a subject-specific frailty with a parametric (Geometric, Poisson, or Negative Binomial) distribution. dos Santos *and others* (1995) used a subject-specific non-parametric frailty, while Guo and Rodriguez (1992) used a piecewise constant baseline and a shared non-parametric frailty. Li *and others* (1998) used a Cox proportional hazard model with a discrete distributed frailty with a two-points support (one fixed at 1 and the other to be estimated).

Discrete frailty models have also been investigated in a Bayesian framework and few authors used Bayesian non-parametric approaches, such as a Polya tree prior for the frailty term (Walker and Mallick, 1997), or a Dirichlet Process prior (Naskar, 2008; Manda, 2011). Among these, only Naskar (2008) used a non-parametric prior form for the baseline.

This article is the first we are aware of that presents a survival model for hierarchical time-to-event data with both a non-parametric discrete shared frailty, and a non-parametric baseline, in a frequentist framework. The method extends the shared frailty Cox model to include a frailty that has a discrete distribution with an unknown number of elements in its support. Thus no prior structure is imposed on either the clustering or the baseline survival. This leads to both a very flexible model, and a probabilistic clustering technique, which we apply to explore heterogeneity between groups of healthcare providers. This is particularly useful in routinely collected datasets which report data for large numbers of individuals, rather than detailed and accurate records for large numbers of covariates. The methods can identify groups with similar characteristics, motivating further investigation of the reasons for their similarity.

We develop a novel EM algorithm for parameter estimation, viewing frailty models as an incomplete data problem, where the observable data are the times-to-event or the censoring times, and the frailty values are the unobservable data. Methods of Louis (1982) and others for computing the information matrix are compared. Identifiability is an issue that can be solved by constraining the mean of the frailty or the cumulative hazard, as discussed by Heckman and Singer (1984). By using a profile likelihood technique as part of this algorithm, we ensure the frailties are identifiable. Selection of the number of clusters is based on Akaike Information Criterion (AIC), Bayesian Information Criterion (BIC), and the approach of Laird (1978) in which clusters are removed when no individuals are classified to them. We also develop an R package to implement our model, available from https://github.com/fgaspe04/discfrail.

In Section 2, we present the mathematical model; the proposed Expectation–Maximization algorithm is described in Section 3; a simulation study provides insights into the scope and limitations of the model in Section 4; while in Section 5 the model is applied to the regional clinical administrative database. Section 6 provides discussion of the results and the future perspectives.

## 2. Semi-parametric Cox model with a non-parametric frailty

Consider a random sample with a hierarchical structure, i.e. where each statistical unit belongs to one group. Define $T_{ij}^*$ as the survival time and $C_{ij}$ as the censoring time of subject $i$, $i = 1,\ldots,n_j$, in the $j$th group, $j=1,\ldots,J$. Let $\boldsymbol{X}_{ij} = (X_{ij1},\ldots, X_{ijp} )^T$ be the vector of covariates, assumed constant over time, for subject $i$ in group $j$. Then, we define $T_{ij}={\rm min}(T_{ij}^*, C_{ij})$, $t_{ij}$ its realization and $\delta_{ij}=\mathbf{1}_{(T_{ij}^* \leq C_{ij} )}$. Let $\tilde{\textbf{w}}$ be the vector of shared random effects, and **w**, $\textbf{w}=\exp{\tilde{\textbf{w}}}$, be the vector of shared frailties. In this work, we introduce a non-parametric frailty term, which can be modeled through a random variable with discrete distribution, with an unknown number of points in the support. In particular, we assume that each group $j$ can belong to one latent population $k$, $k=1,\ldots,K$, with probability $\pi_{k}$. In this case, $w_1,\ldots,w_K$ are the points in the support of $w$, $K$ is the support’s cardinality and $\mathbb{P}\{w=w_k\}=\pi_k$. In order to build the model, we introduce an auxiliary indicator random variable $z_{jk}$ which is equal to $1$ if the $j$th group belongs to the $k$th population, so, considering k as a fixed term, $z_{jk} \overset{i.i.d}{\sim} {\rm Bern}(\pi_{k})$. The requirement $\sum_{k=1}^K z_{jk} = 1$, for each $j$, is equivalent to the assumption that each group belongs to only one population. The vector $\mathbf{z}_{j}$ is distributed as a multinomial. Note that there are two levels of clustering: the first one is known (i.e., healthcare providers as clusters of patients), and we refer to these clusters as groups, while the second level is the unknown clustering of healthcare providers that we want to detect, and we refer to these clusters as latent populations.

The hazard function for individual $i$ in group $j$ conditional on $w_k$ and on $z_{jk}$ is:

(2.1) \begin{equation*}\lambda(t;\boldsymbol{X}_{ij},w_k,z_{jk})=\prod_{k=1}^{K}\left[\lambda_0(t) w_k\exp(\boldsymbol{X}_{ij}^T\boldsymbol{\beta})\right]^{z_{jk}}, \label{eq:hazardnonparam}\end{equation*}

where $\lambda_0(t)$ represents the baseline hazard, $\boldsymbol{\beta}$ is the vector of regression coefficients, and $w_k$ is the frailty term shared among groups of the same latent population $k$. Both the frailty and the baseline hazard are assumed to be non-parametric, which makes model (2.1) an extension of a proportional hazard Cox model. The observable data $\boldsymbol{Y}$ are made up of the set of $\boldsymbol{Y}_{ij}= \{T_{ij},\delta_{ij},\boldsymbol{X}_{ij}\}$ over all $i,j$. We define this as the “incomplete” data, while the “complete” data are the realizations of the vector $\{T_{ij},\delta_{ij},\boldsymbol{X}_{ij},w_k,z_{jk}\}$. We also assume that censoring is non-informative, thus that $T_{ij}^*$ and $C_{ij}$ are conditionally independent, given $\boldsymbol{X}_{ij}$, $w_k$ and $z_{jk}$.

Starting from the hazard rate, we can write down the full likelihood of our model for the complete data explicitly:

(2.2) \begin{eqnarray*}L_{\rm full}(\boldsymbol{\theta};\boldsymbol{Y} | \textbf{z})=\prod_{k=1}^{K} \prod_{j=1}^{J} \pi_k^{z_{jk}} \cdot L_{\rm full}^{jk}(\boldsymbol{\theta}; \boldsymbol{Y}_j | \textbf{z}),\label{eq:fulllik}\end{eqnarray*}

where

(2.3) \begin{eqnarray*}L_{\rm full}^{jk} (\boldsymbol{\theta}; \boldsymbol{Y}_j | \textbf{z})&=&\prod_{i=1}^{n_j}\left\{[\lambda_0(t_{ij}) w_k \exp(X_{ij}^T\beta)] ^{\delta_{ij}} \cdot\exp\left[- \Lambda_0(t_{ij}) w_k \exp(\boldsymbol{X}_{ij}^T\boldsymbol{\beta})\right]\!\right\}^{z_{jk}},\qquad\label{eq:fulllikjth}\end{eqnarray*}

and $\boldsymbol{\theta}=(\boldsymbol{\pi}, \textbf{w}, \lambda_0(t),\boldsymbol{\beta})$ is the vector of parameters, $\textbf{z}:= \{z_{jk}\}_{j=1:J}^{k=1:K}$ is the matrix of random vectors $\{\textbf{z}_{j}\}_{j=1:J}$ indicating membership of groups $j$ in populations $k$, and $\Lambda_0(t) = \int_{0}^{t}\lambda_0(s) {\rm ds}$ is the cumulative baseline hazard function.

This model can be interpreted as a shared frailty Cox model where the frailties are shared among groups of the same latent population, and also as a mixture model, where each component is a survival distribution, $\boldsymbol{\pi}$ is the vector of mixing proportions, and **w** is the vector of component-specific frailties. Finally, the number of latent populations, $K$, can be considered as an unknown parameter, and the relative hazard between two individuals with the same covariate values but from different latent populations $k$ and $k^{\prime}$ can be described by the frailty ratio $w_k/w_{k^{\prime}}$. We note that the model as written is over-parameterized, since the same likelihood would result from multiplying $\lambda_0(t)$ by a constant $c$ while dividing all the $w_k$ by $c$, but identifiability is ensured within the estimation algorithm (Section 3.1).

## 3. Computation

### 3.1. A tailored expectation maximization algorithm

We propose a novel Expectation–Maximization (EM) algorithm (Dempster *and others*, 1977) to estimate $\theta$ for a given $K$. The algorithm iterates between two steps, Expectation and Maximization and, under regularity conditions, the algorithm is guaranteed to converge to a stationary point (Dempster *and others*, 1977).

**E-step:** The full log-likelihood (2.2)–(2.3) can be decomposed into two parts, the first (3.1) depending on $\boldsymbol{\pi}$ and the second (3.2) depending on $\lambda_0(t),\boldsymbol{\beta}, \textbf{w}$.

(3.1) \begin{eqnarray*}&l_{{\rm full},1}(\boldsymbol{\pi}; \boldsymbol{Y} | \textbf{z})=\sum_{k=1}^{K}\sum_{j=1}^{J} z_{jk} \cdot \log(\pi_k).&\label{eq:loglikpi}\\\end{eqnarray*}

(3.2) \begin{eqnarray*}&l_{{\rm full},2}(\lambda_0(t),\boldsymbol{\beta}, \textbf{w}; \boldsymbol{Y} |\textbf{z})= \sum_{k=1}^{K}\sum_{j=1}^{J} z_{jk} \Bigg\{ \cdot\sum_{i=1}^{n_j}\delta_{ij}[\log(\lambda_0(t_{ij})) + \log(w_k) +\boldsymbol{X}_{ij}^T\boldsymbol{\beta}] -\Lambda_0(t_{ij}) w_k\exp(\boldsymbol{X}_{ij}^T\boldsymbol{\beta})\Bigg\}\!.&\label{eq:loglikothers}\nonumber\\\end{eqnarray*}

The Expectation step consists of computing:

$$\begin{eqnarray*}Q(\boldsymbol{\theta})=\mathbb E_{\textbf{z}|\hat{\boldsymbol{\theta}}}[l_{{\rmfull}}(\boldsymbol{\theta}; \boldsymbol{Y}| \textbf{z})] =\mathbb E_{\textbf{z}|\hat{\boldsymbol{\theta}}}[l_{{\rmfull},1}(\boldsymbol{\theta}; \boldsymbol{Y} | \textbf{z})] + \mathbb E_{\textbf{z}|\hat{\boldsymbol{\theta}}}[l_{{\rmfull},2}(\boldsymbol{\theta}; \boldsymbol{Y}| \textbf{z})], \label{eq:Q}\end{eqnarray*}$$

which is the expectation over $\textbf{z}$, given the current values of parameters $\widehat\theta  = (\hat \pi ,{\hat \lambda _0}(t),\widehat\beta ,\widehat{\bf{w}})$, of the full log-likelihood for the observed data $\boldsymbol{Y}$. This reduces to the computation of $\mathbb E[z_{jk}| \boldsymbol{Y}, \hat{\boldsymbol{\theta}}]$, which we then include in (3.1) and (3.2). $\mathbb E[z_{jk}| \boldsymbol{Y}, \hat{\boldsymbol{\theta}}]$ can be derived in closed form using Bayes’ theorem:

$$\begin{eqnarray*}\mathbb E[z_{jk}| \boldsymbol{Y}, \hat{\boldsymbol{\theta}}] = \frac{ \pi_k\exp \left\{\sum_{i=1}^{n_j} \delta_{ij} \cdot \log(w_k) -\Lambda_0(t_{ij}) w_k e^{\boldsymbol{X}_{ij}^T\boldsymbol{\beta}} \right\}}{\sum\limits_{r=1}^K \pi_r \exp \left\{\sum_{i=1}^{n_j} \delta_{ij}\log(w_r) - \Lambda_0(t_{ij}) w_r e^{\boldsymbol{X}_{ij}^T\boldsymbol{\beta}}\right\}}.\label{eq:zjk}\end{eqnarray*}$$

For simplicity, we write $\alpha_{jk}:= \mathbb E[z_{jk}|\boldsymbol{Y}, \hat{\boldsymbol{\theta}}]$, which represents the probability that group $j$ belongs to latent population $k$. Furthermore, we note that this step is similar to the posterior probability computation in general mixture models.

**M-step:** The Maximization step consists of maximizing $Q(\boldsymbol{\theta})$ with respect to $\boldsymbol{\theta}$. $Q(\boldsymbol{\theta})$ can be partitioned so that we can maximize $Q_1(\boldsymbol{\pi}):= \mathbb E_{\textbf{z}|\hat{\boldsymbol{\theta}}}[l_{{\rm full},1}|\boldsymbol{Y},\hat{\boldsymbol{\theta}}]$ with respect to $\boldsymbol{\pi}$ and $Q_2(\lambda_0,\boldsymbol{\beta}, \textbf{w}):=\mathbb E_{\textbf{z}|\hat{\boldsymbol{\theta}}}[l_{{\rm full},2}|\boldsymbol{Y},\hat{\boldsymbol{\theta}}]$ with respect to $\lambda_0,\boldsymbol{\beta}, \textbf{w}$ separately. The maximization of $Q_1(\boldsymbol{\pi})$ is a constrained optimization problem, since $\sum_{k=1}^K\pi_{k}$ is equal to $1$, and we can solve it by applying the Lagrange multipliers technique:

$$\begin{eqnarray*}\hat{\pi}_k = \frac{1}{J}\displaystyle \sum_{j=1}^{J} \alpha_{jk}.\label{eq:bestpi}\end{eqnarray*}$$

The optimization of $Q_2(\lambda_0,\boldsymbol{\beta}, \textbf{w})$ is not trivial, since we adopt a non-parametric baseline hazard. We note that $Q_2(\lambda_0,\boldsymbol{\beta}, \textbf{w})$ is a weighted version of the log-likelihood in a Cox regression model with known offset. Following Johansen (1983) we adapt a profile log-likelihood approach for the estimation of the shared parametric frailty Cox model. Initially, we estimate the $\textbf{w}$ fixing $\lambda_0,\boldsymbol{\beta}$, giving:

$$\begin{equation*}\hat{w}_k = \frac{ \displaystyle \sum_{j=1}^{J} \alpha_{jk}\displaystyle\sum_{i=1}^{n_j} \delta_{ij}}{\displaystyle \sum_{j=1}^{J} \alpha_{jk}\displaystyle \sum_{i=1}^{n_j} \left\{ \Lambda_0(t_{ij}) \cdot \exp(\boldsymbol{X}_{ij}^T\boldsymbol{\beta})\right\}}.\label{eq:westimate}\end{equation*}$$

By substituting these estimates in $Q_2$, we obtain:

(3.3) \begin{equation*}Q_2(\lambda_0,\boldsymbol{\beta},\hat{\textbf{w}})=\sum_{k=1}^{K}\sum_{j=1}^{J}\alpha_{jk} \cdot \Bigg\{ \sum_{i=1}^{n_j}\delta_{ij}[\log(\lambda_0(t_{ij})) + \log(\hat{w}_k) +\boldsymbol{X}_{ij}^T\boldsymbol{\beta}] - \Lambda_0(t_{ij}) \hat{w}_k\exp(\boldsymbol{X}_{ij}^T\boldsymbol{\beta}) \Bigg\}. \label{eq:Q2A}\quad\end{equation*}

We can rewrite $Q_2$ in the following form, recalling that $\sum_{k=1}^K\alpha_{jk}=1$,

$$\begin{eqnarray*}Q_2(\lambda_0,\boldsymbol{\beta},\hat{\textbf{w}}) &=& \sum_{j=1}^{J}\sum_{i=1}^{n_j}\delta_{ij}\log(\lambda_0(t_{ij}))+\delta_{ij}\left(\sum_{k=1}^{K} \alpha_{jk}\log(\hat{w}_k)\right)\nonumber\\&&+\,\delta_{ij}\{\boldsymbol{X}_{ij}^T\boldsymbol{\beta}\} - \Lambda_0(t_{ij})
\left(\sum_{k=1}^{K} \alpha_{jk}\hat{w}_k\right)\exp(\boldsymbol{X}_{ij}^T\boldsymbol{\beta}). \label{eq:Q2}\end{eqnarray*}$$

This is similar to the form of the log-likelihood in a Cox regression model with known offset $\log\left(\sum_{k=1}^{K} \alpha_{jk}\hat{w}_k\right)$. With arguments similar to Johansen (1983), it is possible to show that the estimate of the cumulative baseline that maximizes (3.3) is the following:

(3.4) \begin{eqnarray*}\hat{\Lambda}_0(t_{ij})= \sum_{(fg): t_{fg}\leq t_{ij}}\frac{d_{fg}}{ \sum\limits_{rs\in R(t_{fg})} \left(\sum_{k=1}^{K}\alpha_{sk}\hat{w}_k\right)\exp(\boldsymbol{X}_{rs}^T\boldsymbol{\beta})},\label{eq:NelsonAalen}\end{eqnarray*}

where $d_{fg}$ is the total number of events happening at time $t_{fg}$ and $R(t_{fg})$ represents the set of patients who are at risk at time $t_{fg}$, which is the event time of patient $f$ in cluster $g$.

Including (3.4) in $Q_2(\lambda_0,\boldsymbol{\beta},\hat{\textbf{w}})$, we obtain the profile log-likelihood as a function of only $\boldsymbol{\beta}$:

(3.5) \begin{align*}l_{{\rm profile}}(\boldsymbol{\beta}) = \sum_{j=1}^{J}\sum_{i=1}^{n_j}\delta_{ij}\Bigg[\boldsymbol{X}_{ij}^T\boldsymbol{\beta}-\left. \log\sum_{rs\in R(t_{ij})}\left(\sum_{k=1}^{K} \alpha_{sk}\hat{w}_k\right)\exp(\boldsymbol{X}_{rs}^T\boldsymbol{\beta})\right]\!.\label{eq:lprofile}\end{align*}

Since (3.5) is of the form of the usual partial log-likelihood in the Cox model with known offsets, standard software can be used to obtain the maximum $\hat{\boldsymbol{\beta}}$.

The profile likelihood method also ensures identifiability between $\lambda_0(t)$ and the $w_k$, since at each step of the algorithm, one is estimated conditionally on the current value of the other. We observed better convergence from leaving the $w_k$ unconstrained, compared to applying a constraint such as $w_1 = 1$ in the EM algorithm. However, for interpretability, we divide each estimate of the $w_k$ by the lowest value of $w_k$, obtaining the hazard ratio (HR) for an individual in the $k_{th}$ latent population, compared to an individual with the same characteristics in the lowest-risk latent population. We also investigated order constraints on the $w_k$, though obtained theoretically identical estimates.

To conclude, it is known that when assuming proportional hazards conditionally on a gamma distributed frailty, the ratio of marginal hazards converges monotonely towards one, while it has been proven by Hougaard (1991) that marginal HR for binary mixture of frailties has the same value at time 0 and at infinity, because the frailty mean is the same across groups at these times. Consequently, the marginal HR evolves non-monotonely in time.

### 3.2. Estimation of the standard errors

In the case of the Cox model with shared frailty terms, the variance–covariance matrix can be derived from the observed information matrix $\textbf{I}(\boldsymbol{\theta})^{-1}$Klein (1992), and this has been shown to be a consistent estimator (Parner, 1998). The observed information matrix can be written as:

$$\begin{eqnarray*}\textbf{I}(\boldsymbol{\theta})=-\frac{\partial^{2}l(\boldsymbol{\theta})}{\partial\boldsymbol{\theta}^{2}},\end{eqnarray*}$$

where $l(\boldsymbol{\theta})$ is the observable log-likelihood:

$$\begin{eqnarray*}l(\boldsymbol{\theta}) = \sum_{j=1}^{J}\sum_{i=1}^{n_j}\delta_{ij}\log(\lambda_0(t_{ij})\exp(\boldsymbol{X}_{ij}^T\boldsymbol{\beta})) +\log (\sum_{k=1}^{K} \pi_{k} w_k^{D_j} \cdot\exp\sum_{i=1}^{n_j}\left[-\Lambda_0(t_{ij}) w_k \exp(\boldsymbol{X}_{ij}^T\boldsymbol{\beta})\right]),\end{eqnarray*}$$

where $D_j$ is the total number of events in group $j$, $D_j=\sum_{i=1}^{n_j}\delta_{ij}$. Note that this is obtained by integrating the full likelihood over the random variable $\textbf{z}$. For further information about the derivation of the observable log-likelihood and the elements of the observed information matrix, see Appendix A of supplementary material available at *Biostatistics* Online.

This asymptotic estimate of the covariance matrix can be computed once the parameters are estimated from the EM algorithm. A more computationally convenient approximation that exploits the EM framework was proposed by Louis (1982) together with a method to accelerate the algorithm, and a proof of quadratic convergence near the maximum likelihood estimate. Louis (1982) states that the $j$th component of the observed information matrix **I** can be written as:

$$\begin{eqnarray*}\textbf{I}^j=\mathbb{E}[B_j] - \mathbb{E}[S_j S_j^T] + S_j^{\star}S_j^{\star T},\label{observation_matrix}\end{eqnarray*}$$

where $S$ and $S^{\star}$ are the gradient vectors of the full log-likelihood and the observable log-likelihood respectively, while $B$ is the negative second derivative matrix of the full log-likelihood (see Appendix A of supplementary material available at *Biostatistics* Online for element-wise computation).

In this work, we implement both methods and we compare the obtained results (see Table 1 in Section 5). We provide also a third estimate for the observed information matrix, by using numerical methods to obtain the first and second derivatives of the full log-likelihood (Gilbert and Varadhan, 2016).

**Table 1.** Estimates of Cox model with a non-parametric frailty term and a classical Cox model. Std.Err: standard errors

	Cox with non-parametric frailty				Cox model		Cox model with gammafrailty	
Parameters	Estimates	Std. Err.			Estimates	Std. Err.	Estimates	Std. Err.
		Louis	Exact	Numerical				
$\pi_1$	0.47	0.0592	0.0592	0.0593	—	—	—	—
$\pi_2$	0.53	—	—	—	—	—	—	—
$w_2/w_1$	1.40	0.0343	0.0343	0.0343	—	—	—	—
Variance (frailty gamma)	—	—	—	—	—	—	0.04	—
Log-hazard ratios								
10 years of age	0.171	8.57 $10^{-4}$	8.57 $10^{-4}$	8.57 $10^{-4}$	0.169	8.57 $10^{-4}$	0.172	8.61 $10^{-4}$
Male	0.223	0.0181	0.0181	0.0181	0.229	0.0180	0.221	0.0181
${\geq}3$ comorbidities	0.279	0.0176	0.0176	0.0176	0.279	0.0174	0.277	0.0177
Number of procedures	$-$0.091	0.0126	0.0126	0.0126	$-$0.089	0.0125	$-$0.089	0.0127
Log-lik	$-$137,186.3				$-$137,364.7		$-$137,255.9	

In this work, we estimate the frailties separately; however, because we are interested in the ratios of frailties, we estimate the standard errors related to the ratios through the following formula:

$$\begin{eqnarray*}{\rm Var}(\hat{w}_k/\hat{w}_1) =\left(\frac{\mu_{\hat{w}_k}}{\mu_{\hat{w}_1}}\right)^2\cdot\left[\frac{\sigma_{\hat{w}_1}^2}{\mu_{\hat{w}_1}^2}+\frac{\sigma_{\hat{w}_k}^2}{\mu_{\hat{w}_k}^2}-\frac{2{\rmCov}(\hat{w}_1,\hat{w}_k)}{\mu_{\hat{w}_1}\mu_{\hat{w}_k}}\right]\!,\end{eqnarray*}$$

which can be derived by using the first and second order Taylor expansions.

### 3.3. Selection of the number of latent populations

Since it is not possible to estimate $K$ using a log-likelihood maximization argument (Figueiredo and Jain, 2002) we estimate $\boldsymbol{\theta}$ for each potential $K$, and compute a model selection criterion such as AIC, BIC, or search for the optimal $K$ using the approach proposed by Laird (1978). At the end of the analysis, each provider $j$ is assigned to that latent population $k$ for which, $k = \mathop {{\rm{arg}}{\rm{max}}}\limits_{k = 1:K} {\alpha _{jk}}$. For all the computations, we used the R software (R Development Core Team, 2016) developing an R package available from https://github.com/fgaspe04/discfrail.

## 4. Simulation study

A simulation study was conducted to evaluate the performance of the estimators obtained with the algorithm described in Section 3. We simulated 1000 datasets, each with $J=100$ groups (e.g., healthcare providers), and $n_j=50$ statistical units (e.g., patients) per group, giving a total of $5000$ records in each dataset. For all simulations, we set the covariate-related log HR $\boldsymbol{\beta}=0.4$, and define the baseline cumulative hazard so that $\Lambda_0(t)= (100 \cdot t)^{1/1.9}$ ($\Lambda_0^{-1}(t)= 0.01 \cdot t^{1.9}$) in order to mimic the dataset that motivated this work. The aim of the simulation was to estimate how well the algorithm estimates the true frailty ratios $\textbf{w}/w_1$, mixing proportions $\boldsymbol{\pi}$ and number of latent populations $K$ for various values of these parameters of interest.

(i) Firstly we focus on $\boldsymbol{\pi}$, by setting $K=2$ and $w_2/w_1 = 1.55$, and run 9 scenarios with $\pi_1 \in \{0.1,0.2,0.3,0.4,0.5,0.6,0.7,0.8,0.9\}$. The results are shown in Appendix B of supplementary material Available at *Biostatistics Online*. In general, we observe estimates closer to the true values when the mixing proportion, $\pi_1$, is closer to $0.5$, i.e., when there is a relatively large amount of data in both mixture components.
(ii) To assess the effect of a smaller sample of units, we repeat the scenario in (i) with $n_j=35$ statistical units per group, giving a total of $3500$ records in each dataset. The results are shown in Appendix C of supplementary material Available at *Biostatistic* Online and are close to the ones obtained in Appendix B of supplementary material Available at *Biostatistic* Online. This proves a certain robustness of the proposed method with respect to groups’ size.
(iii) We focus on $w_2/w_1$, by setting $K=2$, $\boldsymbol{\pi} = [0.3, 0.7]$ and run 7 scenarios, with $w_2/w_1 \in \{1.14, 1.29, 1.43, 1.57, 1.71, 2, 3\}$. We assume that frailty ratios smaller than 1.1 are not of practical interest. Conversely, we assume that for frailty ratios bigger than about $3$, the presence of two latent populations can be identified easily by exploratory analysis, e.g., plotting a set of survival curves by group. The results are shown in Appendix D of supplementary material Available at *Biostatistic* Online. The estimates of all parameters become more accurate as the frailty ratio increases, thus the contrast between latent populations becomes larger. In particular, the true number of latent populations $K=2$ is detected for values of $w_2/w_1$ of around $1.6$ and higher.
(iv) We focus on $K$, which leads to a complex pattern of simulations since varying $K$ changes the length of the vectors $\boldsymbol{\pi}$ and of $\textbf{w}/w_1$. We tested $K \in \{1,2,3,4\}$, $\pi \in \{ 1, (0.4,0.6), (0.2,0.3,0.5),$$(0.15,0.25,0.3,0.3)\}$ and $\textbf{w}/w_1 \in \{1, (1.5), (1.5, 2.5), (1.5,2.5,4)\}$, respectively. In our application, Section 5, we did not detect more than four populations with either BIC or AIC. The results are shown in Appendix E of supplementary material Available at *Biostatistic* Online. The frailty ratios and mixing proportions are estimated accurately for all values of $K$. However, the three model selection methods produce different estimates of $K$, with BIC recovering the true values more often, and AIC and the method of Laird (1978) tending to estimate higher values.

AIC theoretically favors more complex models than BIC; however, they produced the same estimate for the number of latent populations in the majority of the scenarios. As discussed by Burnham and Anderson (2003), BIC would be preferred if we believe there is a low-dimensional “true” clustering structure which would not change with the amount of data, whereas AIC is preferred if we expect more latent populations (with more weakly contrasting frailties) to be revealed as the dataset becomes bigger. We are unaware of any formal comparison between AIC (or BIC) and the method proposed by Laird (1978).

Overall, the algorithm performs well, especially when there is a moderately large contrast between the frailty in different latent populations, and there is sufficient information in all latent populations. Thus, more clearly defined latent population structures are revealed more easily.

## 5. An application to healthcare structures admission for patients with heart failure

The non-parametric frailty Cox model was applied to administrative data from patients with HF treated in the Lombardia Region, Italy. HF is a chronic disease that is the most common cause of hospitalization in Western countries for people more than $65$ years old, with a 5-year risk of death similar or worse than that observed after a diagnosis of cancer (Frigerio *and others*, 2017).

While the full history of hospital admission and death due to HF has been investigated using multi-state models (Gasperoni *and others*, 2017), an event of particular interest is the first readmission, as a marker of success of the initial treatment and possible future health care use. The time to readmission is thought to be particularly related to healthcare provider policies, infrastructure, extent or expertise of staff, efficiency or case mix. These unobserved covariates may cause over-dispersion. By assuming a shared frailty between patients admitted to the same healthcare provider, we investigate how the time to first readmission is associated with the healthcare provider. Furthermore, we use the non-parametric frailty distribution to detect clusters of healthcare providers with similar outcomes.

In our first analysis we included the 121 994 patients who had a first admission for HF between $2005$ and $2010$. The outcome was defined as the time between the first discharge and the second admission. We consider those patients who died in this interval as censored, which ignores the fact that death and readmission are competing events, but leads to lower bias than if we were to exclude patients who died in this interval. Moreover, we included only healthcare providers with more than $20$ patients.

Our model identified three latent populations using the BIC for selection, four with AIC and six with Laird’s criterion (Laird, 1978). This result is in line with the simulations, since BIC penalizes complex models and Laird (1978) tended to overestimate the number of latent populations. In the case of three latent populations, we estimate $\boldsymbol{\pi} = [0.19, 0.43, 0.38]$ and $\textbf{w}/w_1 = [1, 1.78 ,2.26]$. However, the number of patients in this dataset meant that the standard errors could not be computed numerically, although the approximate and exact standard errors could be computed. Therefore, in order to show the results of the proposed model and method in their entirety, we focused on a smaller dataset which included only patients whose first discharge was recorded between $2006$ and $2007$. The reduced dataset included $40\,337$ patients from $138$ healthcare providers. This sample had an average age of 76 years (s.d. 11.7) and $49.7\%$ were male. 44.9% had three or more comorbidities, which include renal disease, tumors, and diabetes. 16.3% of the patients underwent one or more (up to 5) procedures, including coronary artery bypass graft surgery, percutaneous transluminal coronary angioplasty, or insertion of an implantable cardioverter-defibrillator. We applied the non-parametric frailty Cox model, described in Section 2, with four individual-level predictors: age, gender, presence of three or more comorbidities, and the total number of procedures. We fitted models with values of $K$ ranging from $1$ to $7$. The AIC and BIC were optimized by a model with $K=2$ latent populations, while the criterion of Laird (1978) suggested a greater number $K=6$. Since our simulations suggested that where the true $K \leq 4$ and between-population frailty ratios are $>1.1$, AIC and BIC estimate $K$ more accurately, we present the results for $K=2$.

**Fig. 1.**
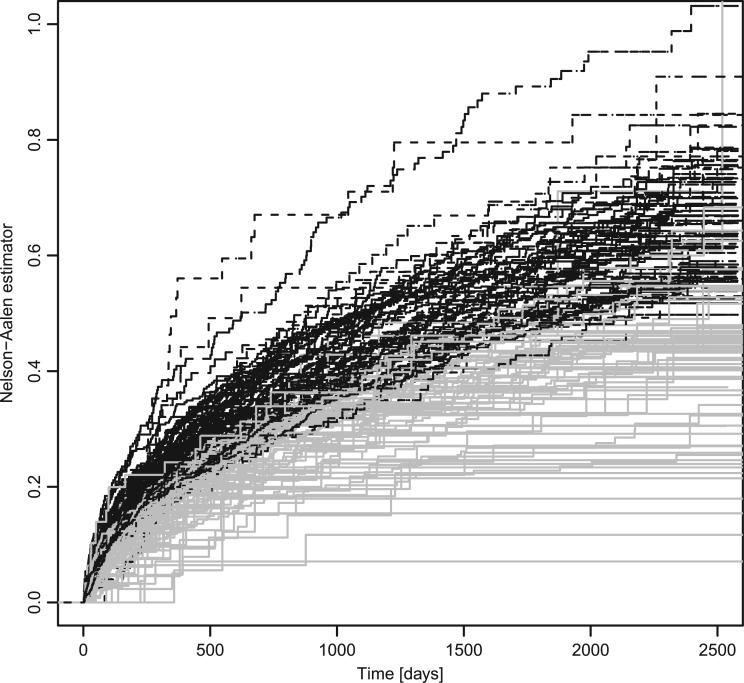
$138$ structure-specific Nelson–Aalen curves drawn according to the membership of two latent populations from the frailty model for time to readmission. The gray curves are related to latent population 1, while the black dashed curves are related to latent population 2.

We plot 138 Nelson–Aalen curves (one for each provider) colored according to the $K=2$ populations identified (Figure 1). A total of 68 providers are assigned to the cluster with lowest risk (gray curves) and $\hat{\pi}_1 = 0.47$. The black curves represent the $\hat{\pi}_2 = 0.53 \%$ of healthcare providers in latent population $2$ with $\hat{w}_2$ = 1.40 times the hazard of readmission relative to $\hat{w}_1$. All estimates for this reduced cohort are reported in Table 1, together with the estimates from a standard Cox model and a Cox model with a Gamma frailty, fitted with $\texttt{frailtyEM}$ (Balan and Putter, 2017). The discrete frailty model describes the data best, as judged by the maximized likelihood values.

There is a small discrepancy between the population classification according to the criterion in Section 3.3, (68 and 70 providers in latent population 1 and 2, respectively) and the result of $\pi \times J$, through which we estimate 65 and 73 providers in latent populations 1 and 2. This is related to the few providers whose probabilities of belonging to population 1 or 2 are close to 0.5 and these providers may require further investigation.

We note that the discrete frailty model has higher log-likelihood than either the standard Cox model or a model with a continuous Gamma frailty (see Table 1). However, patient-level covariate coefficient estimates and standard errors are similar in these 3 models; older people have a higher risk of being readmitted (HR, HR, ${\rm e}^{0.0171}=1.02$ per year of age), as do men (HR ${\rm e}^{0.223}=1.25$), people having three or more comorbidities (HR ${\rm e}^{0.279}=1.32$), and people having fewer medical or surgical procedures (HR ${\rm e}^{-0.091}=0.91$ per procedure). The relationship between fewer procedures and risk of readmission may seem counter-intuitive, but it reflects the fact that people undergoing procedures are younger on average (with mean age 70.3 (11.8), compared to 77.2 (11.4) for people who do not), and there may be some collinearity between age and number of procedures. Moreover, the procedures may have successfully treated the underlying heart disease, thereby reducing the need for readmission.

**Fig. 2.**
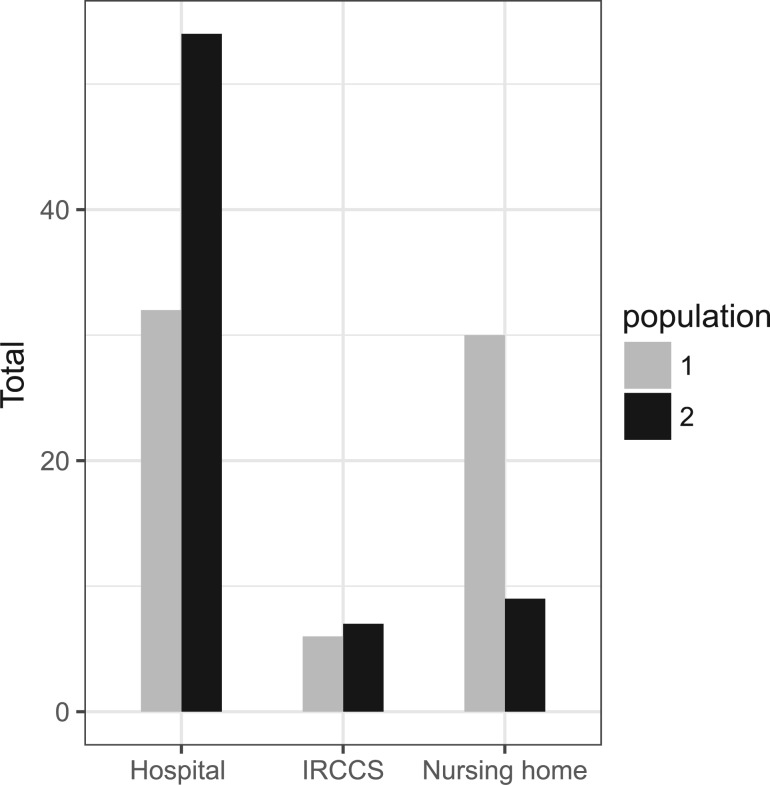
Healthcare providers structures in the two latent populations. Black bars are related to structures that are assigned to the second latent population, while gray bars to the first one.

We then sought to describe the latent population structure, indicated by the model with $K=2$, in terms of characteristics of the healthcare providers that are recorded in the database. Healthcare providers predicted to be in the population with higher risk of readmission, on average, had a higher number of patients and a higher percentage of in-structure deaths per year, although the percentages of surgical and complex cases were similar between the two latent populations, Table 2. Comparing the type of institution, we found that hospitals belonged to the higher-frailty population more often, while nursing homes tended to belong to the lower-frailty population, Figure 2.

**Table 2.** Profiling of healthcare providers assigned to the detected latent populations. s.d.: standard deviation

	Latent population 1	Latent population 2
Number of providers	68	70
Average number of patients (s.d.)	6581.1 (6 203.1)	12 147.4 (9989.3)
Average $\%$ of in-structure death (s.d.)	3.29 (3.08)	3.61 (1.81)
Average $\%$ of surgical cases (s.d.)	30.75 (21.40)	30.18 (12.44)
Average $\%$ of complex cases (s.d.)	14.03 (5.63)	14.52 (3.40)

We then extended the frailty model to include two structure-specific covariates, describing the type of healthcare provider with three categories, as in Figure 2, and the percentage of admissions in which the patient died. The optimal model according to AIC and BIC still has $K=2$, with relative frailty $w_2/w_1=1.43$ between the two populations, and an estimated probability of $\pi_2=0.64$ that the healthcare provider belongs to the group with the higher frailty. Thus these two covariates can characterize the two latent populations only partially, and the remaining clustering pattern probably depends on unobserved characteristics of the healthcare providers. The non-parametric frailty model therefore serves as a starting point for further investigation of the effect of healthcare providers and their characteristics on patient outcomes.

## 6. Discussion

In this article, we propose a new model that deals with hierarchical time-to-event data and tackles two issues: extending the classical Cox proportional hazard model and detecting a clustering structure among groups by including a shared non-parametric frailty term. Classical approaches for hierarchical time-to-event data use proportional hazard models with a parametric shared frailty; however, the most appropriate parametric frailty distribution will not always be clear and the data may not fit any standard parametric family (Austin, 2017). Having a discrete frailty distribution, together with an unspecified baseline hazard, leads to a relatively novel and very flexible model for grouped survival data.

Moreover, we are able to detect clustering at the second level of a hierarchy of time-to-event data. Indeed, we can identify the existence and nature of a clustering structure, without defining *a priori* a set of covariates that describe the investigators’ opinions about the performance of the healthcare providers. A further strength is that it may be used to detect clusters of individuals, as well as clusters of groups, since the frailties may be group-related or individual-related. Additionally, detecting more complex hierarchical structures (e.g., patients grouped in structures grouped in regions) may be of interest. In this case, we could consider an extension to nested frailty models, in a frequentist or Bayesian framework.

Usually, the software used to estimate the parameters of proportional hazard models with shared frailties relies on some version of the EM algorithm. In this work, we proposed an EM algorithm that was designed for our model. Other techniques have been explored for specific models: for example, the penalized partial likelihood approach (Therneau and Grambsch, 2000, Section 6, Chapter 9) has been applied for Gamma-distributed frailties (with the same results as the EM) or log-Normal-distributed frailties (with similar results to the EM). Li *and others* (1998) proposed Monte Carlo EM, in which the expectation step is computed through a Monte Carlo simulation. We note that care should be taken to ensure that a global maximum is located when applying this procedure, both by exploring different initial values and by inspection of profile likelihoods. Further extension of our work to investigate alternative implementation methods that could speed up the procedure would be worthwhile. A step in this direction was made by the choice of standard error matrix computation methods. Indeed, we started implementing the numerical approximation of the full log-likelihood through $\texttt{numderiv}$ package (Gilbert and Varadhan, 2016) and, when it turned out to be too computationally heavy and inappropriate for big datasets, we decided to explore two different methods: Louis (1982)’s approximate and exact methods. It is important to note that the computational limitations of the numerical method did not cause a problem, since we are able to estimate the standard errors with both the other two methods (see Section 5). However, several improvements in computational efficiency can be investigated and they would have a significant effect on the analysis of very large databases, such as the administrative clinical database that motivated this work. Such administrative databases are emerging as powerful tools for addressing questions in epidemiology and other medical research; the need for rigorously defined models and reliable methods of analysis is clear. The proposed model, which makes few assumptions about the baseline hazard or frailty distribution, represents a step in this direction. Further extension of this model to a realistic but more complex framework, such as multiple events, would be a natural next step.

## Supplementary Material

kxy071_Supplementary_Materials

## 7. Software

Software in the form of R package is available at https://github.com/fgaspe04/discfrail.git and simulations are available on request from the corresponding author (francesca.gasperoni@polimi.it).
