# Supplementary material for: Non-parametric frailty Cox models for hierarchical time-to-event data
Source: Biostatistics. 2018 Dec 26;21(3):531–44. doi: 10.1093/biostatistics/kxy071 (PMC6451633; doi:10.1093/biostatistics/kxy071)
Supplement: kxy071_Supplementary_Materials [file kxy071_supplementary_materials.pdf]

Biostatistics (2018), **0**, 0, *pp.* 1–15
doi:10.1093/biostatistics/SupplMat

Nonparametric frailty Cox models for hierarchical time-to-event data
Supplementary Materials

FRANCESCA GASPERONI*, FRANCESCA IEVA, ANNA MARIA PAGANONI,

MOX - Modelling and Scientific Computing, Department of Mathematics Politecnico di Milano, piazza Leonardo Da Vinci 32, Milano 20123, Italy

CHRISTOPHER JACKSON

MRC Biostatistics Unit, Cambridge Institute of Public Health, Forvie Site, Robinson Way,Cambridge Biomedical Campus, Cambridge CB2 0SR, U.K.

LINDA SHARPLES

Department of Medical Statistics, London School of Hygiene & Tropical Medicine, Keppel Street, London WC1E 7HT, U.K.

francesca.gasperoni@polimi.it

A. Observed information matrix

In Appendix A we compute the observed information matrix and we compute it in two ways: evaluating the derivatives of the observable loglikelihood and with the Louis method.

*To whom correspondence should be addressed.

© The Author 2018. Published by Oxford University Press. All rights reserved. For permissions, please e-mail: journals.permissions@oup.com

Hessian of the observable loglikelihood

First of all, we write the observable likelihood, which is obtained by integrating out the random variable $\mathbf{z}$:

$$
\begin{aligned}
l(\boldsymbol{\theta}; data) =& \log\left(\prod_{j=1}^{J}\sum_{k=1}^{K}\pi_k\prod_{i=1}^{n_j}\left\{[\lambda_0(t_{ij})w_k\exp\left(\mathbf{X}_{ij}^T\boldsymbol{\beta}\right)]^{\delta_{ij}}\cdot\exp\left[-\Lambda_0(t_{ij})w_k\exp\left(\mathbf{X}_{ij}^T\boldsymbol{\beta}\right)\right]\right\}\right) = \\
&\sum_{j=1}^{J} log\left(\sum_{k=1}^{K}\pi_k\prod_{i=1}^{n_j}\left\{[\lambda_0(t_{ij})w_k\exp\left(\mathbf{X}_{ij}^T\boldsymbol{\beta}\right)]^{\delta_{ij}}\cdot\exp\left[-\Lambda_0(t_{ij})w_k\exp\left(\mathbf{X}_{ij}^T\boldsymbol{\beta}\right)\right]\right\}\right) = \\
&\sum_{j=1}^{J} log\left[\prod_{i=1}^{n_j}[\lambda_0(t_{ij})\exp\left(\mathbf{X}_{ij}^T\boldsymbol{\beta}\right)]^{\delta_{ij}}\cdot\left(\sum_{k=1}^{K}\pi_k w_k^{D_j}\cdot\prod_{i=1}^{n_j}\exp\left[-\Lambda_0(t_{ij})w_k\exp\left(\mathbf{X}_{ij}^T\boldsymbol{\beta}\right)\right]\right)\right] = \\
&\sum_{j=1}^{J}\sum_{i=1}^{n_j}\delta_{ij}\log\left(\lambda_0(t_{ij})\exp\left(\mathbf{X}_{ij}^T\boldsymbol{\beta}\right)\right) + \log\left(\sum_{k=1}^{K}\pi_k w_k^{D_j}\cdot\exp\sum_{i=1}^{n_j}\left[-\Lambda_0(t_{ij})w_k\exp\left(\mathbf{X}_{ij}^T\boldsymbol{\beta}\right)\right]\right) = \\
&\sum_{j=1}^{J} l_1^j + l_2^j
\end{aligned}
$$

where D_j is the total events happened in group j, $D_j = \sum_{i=1}^{n_j}\delta_{ij}$.

$$
l_1^j = \sum_{i=1}^{n_j}\delta_{ij}\log\left(\lambda_0(t_{ij})\exp\left(\mathbf{X}_{ij}^T\boldsymbol{\beta}\right)\right)
$$

$$
l_2^j = \log\left(\sum_{k=1}^{K}\pi_k w_k^{D_j}\cdot\exp\sum_{i=1}^{n_j}\left[-\Lambda_0(t_{ij})w_k\exp\left(\mathbf{X}_{ij}^T\boldsymbol{\beta}\right)\right]\right)
$$

To compute the second derivatives with respect to all parameters, recall the definition of the baseline and cumulative baseline hazard estimates and the related derivatives:

$$
\lambda_0(t_{fg}) = \frac{d_{fg}}{\sum_{rs\in R(t_{fg})}\left(\sum_{k=1}^{K}\alpha_{sk}w_k\right)\exp\left(\mathbf{X}_{rs}^T\boldsymbol{\beta}\right)} \tag{A.1a}
$$

$$
\lambda_{0\alpha}(t_{fg}) = \frac{\partial\lambda_0(t_{fg})}{\partial\boldsymbol{\beta}_\alpha} = \frac{-d_{fg}\cdot\sum_{rs\in R(t_{fg})}\left(\sum_{k=1}^{K}\alpha_{sk}w_k\right)\exp\left(\mathbf{X}_{rs}^T\boldsymbol{\beta}\right)\mathbf{X}_{rs\alpha}}{\left(\sum_{rs\in R(t_{fg})}\left(\sum_{k=1}^{K}\alpha_{sk}w_k\right)\exp\left(\mathbf{X}_{rs}^T\boldsymbol{\beta}\right)\right)^2} \tag{A.1b}
$$

$$
\begin{aligned}
\lambda_{0\alpha\gamma}(t_{fg}) = \frac{\partial^2\lambda_0(t_{fg})}{\partial\boldsymbol{\beta}_\alpha\partial\boldsymbol{\beta}_\gamma} = \frac{-d_{fg}\cdot\sum_{rs\in R(t_{fg})}\left(\sum_{k=1}^{K}\alpha_{sk}w_k\right)\exp\left(\mathbf{X}_{rs}^T\boldsymbol{\beta}\right)\mathbf{X}_{rs\alpha}\mathbf{X}_{rs\gamma}}{\left(\sum_{rs\in R(t_{fg})}\left(\sum_{k=1}^{K}\alpha_{sk}w_k\right)\exp\left(\mathbf{X}_{rs}^T\boldsymbol{\beta}\right)\right)^2} \\
+\frac{2\cdot d_{fg}\left(\sum_{rs\in R(t_{fg})}\left(\sum_{k=1}^{K}\alpha_{sk}w_k\right)\exp\left(\mathbf{X}_{rs}^T\boldsymbol{\beta}\right)\mathbf{X}_{rs\alpha}\right)\left(\sum_{rs\in R(t_{fg})}\left(\sum_{k=1}^{K}\alpha_{sk}w_k\right)\exp\left(\mathbf{X}_{rs}^T\boldsymbol{\beta}\right)\mathbf{X}_{rs\gamma}\right)}{\left(\sum_{rs\in R(t_{fg})}\left(\sum_{k=1}^{K}\alpha_{sk}w_k\right)\exp\left(\mathbf{X}_{rs}^T\boldsymbol{\beta}\right)\right)^3}
\end{aligned} \tag{A.1c}
$$

$$
\Lambda_{0\alpha}(t_{ij}) = \sum_{fg:t_{fg}\leqslant t_{ij}}\frac{\partial\lambda_0(t_{fg})}{\partial\boldsymbol{\beta}_\alpha} \tag{A.1d}
$$

$$
\Lambda_{0\alpha\gamma}(t_{ij}) = \sum_{fg:t_{fg}\leqslant t_{ij}}\frac{\partial^2\lambda_0(t_{fg})}{\partial\boldsymbol{\beta}_\alpha\partial\boldsymbol{\beta}_\gamma} \tag{A.1e}
$$

where d_{fg} is the total number of events recorded at time t_{fg}.

$$\frac{\partial^2 l_1^j}{\partial \boldsymbol{\beta}_\alpha \partial \boldsymbol{\beta}_\gamma} = \sum_{i=1}^{n_j} \delta_{ij} \left\{ \frac{\lambda_{0\alpha\gamma}(t_{ij})\lambda_0(t_{ij}) - \lambda_{0\alpha}(t_{ij})\lambda_{0\gamma}(t_{ij})}{\lambda_0(t_{ij})^2} \right\}, \quad \alpha, \gamma = 1:p$$

where p is the total number of covariates.

$$\frac{\partial l_2^j}{\partial \pi_g} = \frac{w_g^{D_j} \exp\{-w_g \sum_{i=1}^{n_j} \Lambda_0(t_{ij}) \exp\left(\mathbf{X}_{ij}^T \boldsymbol{\beta}\right)\} - w_K^{D_j} \exp\{-w_K \sum_{i=1}^{n_j} \Lambda_0(t_{ij}) \exp\left(\mathbf{X}_{ij}^T \boldsymbol{\beta}\right)\}}{\sum_{k=1}^K \pi_k w_k^{D_j} \cdot \exp\{-w_k \sum_{i=1}^{n_j} \Lambda_0(t_{ij}) \exp\left(\mathbf{X}_{ij}^T \boldsymbol{\beta}\right)\}}, \quad g = 1:(K-1)$$

$$\frac{\partial l_2^j}{\partial w_q} = \frac{\pi_q w_q^{D_j - 1} \exp\{-w_q \sum_{i=1}^{n_j} \Lambda_0(t_{ij}) \exp\left(\mathbf{X}_{ij}^T \boldsymbol{\beta}\right)\} \left(D_j - w_q \sum_{i=1}^{n_j} \Lambda_0(t_{ij}) \exp\left(\mathbf{X}_{ij}^T \boldsymbol{\beta}\right)\right)}{\sum_{k=1}^K \pi_k w_k^{D_j} \cdot \exp\{-w_k \sum_{i=1}^{n_j} \Lambda_0(t_{ij}) \exp\left(\mathbf{X}_{ij}^T \boldsymbol{\beta}\right)\}}, \quad q = 1:K$$

$$\frac{\partial l_2^j}{\partial \boldsymbol{\beta}_\alpha} = \frac{-\sum_{k=1}^K \pi_k w_k^{D_j+1} \exp\{-w_k \sum_{i=1}^{n_j} \Lambda_0(t_{ij}) \exp\left(\mathbf{X}_{ij}^T \boldsymbol{\beta}\right)\} \sum_{i=1}^{n_j} (\Lambda_{0\alpha}(t_{ij}) + \Lambda_0(t_{ij}) X_{ij\alpha}) \exp\{\mathbf{X}_{ij}^T \boldsymbol{\beta}\}}{\sum_{k=1}^K \pi_k w_k^{D_j} \cdot \exp\{-w_k \sum_{i=1}^{n_j} \Lambda_0(t_{ij}) \exp\left(\mathbf{X}_{ij}^T \boldsymbol{\beta}\right)\}}, \quad \alpha = 1:p$$

$$\begin{aligned}\frac{\partial^2 l_2^j}{\partial \pi_g \partial \pi_l} &= \frac{-\left(w_g^{D_j} \exp\{-w_g \sum_{i=1}^{n_j} \Lambda_0(t_{ij}) \exp\left(\mathbf{X}_{ij}^T \boldsymbol{\beta}\right)\} - w_K^{D_j} \exp\{-w_K \sum_{i=1}^{n_j} \Lambda_0(t_{ij}) \exp\left(\mathbf{X}_{ij}^T \boldsymbol{\beta}\right)\}\right)}{\left(\sum_{k=1}^K \pi_k w_k^{D_j} \cdot \exp\{-w_k \sum_{i=1}^{n_j} \Lambda_0(t_{ij}) \exp\left(\mathbf{X}_{ij}^T \boldsymbol{\beta}\right)\}\right)^2} \cdot \\ &\cdot \left(w_l^{D_j} \exp\{-w_l \sum_{i=1}^{n_j} \Lambda_0(t_{ij}) \exp\left(\mathbf{X}_{ij}^T \boldsymbol{\beta}\right)\} - w_K^{D_j} \exp\{-w_K \sum_{i=1}^{n_j} \Lambda_0(t_{ij}) \exp\left(\mathbf{X}_{ij}^T \boldsymbol{\beta}\right)\}\right), \quad g, l = 1:(K-1)\end{aligned}$$

$$\begin{aligned}\frac{\partial^2 l_2^j}{\partial w_q \partial w_r} &= \mathbf{1}_{\{r=q\}} \left\{ \frac{\pi_q w_q^{D_j - 2} \exp\{-w_q \sum_{i=1}^{n_j} \Lambda_0(t_{ij}) \exp\left(\mathbf{X}_{ij}^T \boldsymbol{\beta}\right)\}}{\sum_{k=1}^K \pi_k w_k^{D_j} \cdot \exp\{-w_k \sum_{i=1}^{n_j} \Lambda_0(t_{ij}) \exp\left(\mathbf{X}_{ij}^T \boldsymbol{\beta}\right)\}} \cdot \right. \\ &\left. \left[\left(D_j - 1 - w_q \sum_{i=1}^{n_j} \Lambda_0(t_{ij}) \exp\left(\mathbf{X}_{ij}^T \boldsymbol{\beta}\right)\right) \left(D_j - w_q \sum_{i=1}^{n_j} \Lambda_0(t_{ij}) \exp\left(\mathbf{X}_{ij}^T \boldsymbol{\beta}\right)\right) - w_q \sum_{i=1}^{n_j} \Lambda_0(t_{ij}) \exp\left(\mathbf{X}_{ij}^T \boldsymbol{\beta}\right) \right] \right\} \\ &- \frac{\pi_g \pi_l (w_q w_r)^{D_j - 1} \exp\{-(w_q + w_r) \sum_{i=1}^{n_j} \Lambda_0(t_{ij}) \exp\left(\mathbf{X}_{ij}^T \boldsymbol{\beta}\right)\}}{\left(\sum_{k=1}^K \pi_k w_k^{D_j} \cdot \exp\{-w_k \sum_{i=1}^{n_j} \Lambda_0(t_{ij}) \exp\left(\mathbf{X}_{ij}^T \boldsymbol{\beta}\right)\}\right)^2} \cdot \\ &\cdot \left(D_j - w_q \sum_{i=1}^{n_j} \Lambda_0(t_{ij}) \exp\left(\mathbf{X}_{ij}^T \boldsymbol{\beta}\right)\right) \left(D_j - w_r \sum_{i=1}^{n_j} \Lambda_0(t_{ij}) \exp\left(\mathbf{X}_{ij}^T \boldsymbol{\beta}\right)\right), \quad q, r = 1:K\end{aligned}$$

$$
\begin{aligned}
\frac{\partial^2 l_2^j}{\partial \boldsymbol{\beta}_\alpha \partial \boldsymbol{\beta}_\gamma} = & \frac{-\sum_{k=1}^K \pi_k w_k^{D_j+1} \exp\{-w_k \sum_{i=1}^{n_j} \Lambda_0(t_{ij}) \exp\left(\mathbf{X}_{ij}^T \boldsymbol{\beta}\right)\}}{\sum_{k=1}^K \pi_k w_k^{D_j} \cdot \exp\{-w_k \sum_{i=1}^{n_j} \Lambda_0(t_{ij}) \exp\left(\mathbf{X}_{ij}^T \boldsymbol{\beta}\right)\}} \cdot \\
& \cdot \Bigg\{ -w_k \sum_{i=1}^{n_j} (\Lambda_{0\alpha}(t_{ij}) + \Lambda_0(t_{ij}) X_{ij\alpha}) \exp\{\mathbf{X}_{ij}^T \boldsymbol{\beta}\} \cdot \sum_{i=1}^{n_j} (\Lambda_{0\gamma}(t_{ij}) + \Lambda_0(t_{ij}) X_{ij\gamma}) \exp\{\mathbf{X}_{ij}^T \boldsymbol{\beta}\} + \\
& + \sum_{i=1}^{n_j} (\Lambda_{0\alpha\gamma}(t_{ij}) + \Lambda_{0\gamma}(t_{ij}) X_{ij\alpha} + \Lambda_{0\alpha}(t_{ij}) X_{ij\gamma} + \Lambda_0(t_{ij}) X_{ij\gamma} X_{ij\alpha}) \exp\{\mathbf{X}_{ij}^T \boldsymbol{\beta}\} \Bigg\} + \\
& - \frac{(\sum_{k=1}^K \pi_k w_k^{D_j+1} \exp\{-w_k \sum_{i=1}^{n_j} \Lambda_0(t_{ij}) \exp\left(\mathbf{X}_{ij}^T \boldsymbol{\beta}\right)\})^2}{\left(\sum_{k=1}^K \pi_k w_k^{D_j} \cdot \exp\{-w_k \sum_{i=1}^{n_j} \Lambda_0(t_{ij}) \exp\left(\mathbf{X}_{ij}^T \boldsymbol{\beta}\right)\}\right)^2} \cdot \\
& \cdot \sum_{i=1}^{n_j} (\Lambda_{0\alpha}(t_{ij}) + \Lambda_0(t_{ij}) X_{ij\alpha}) \exp\{\mathbf{X}_{ij}^T \boldsymbol{\beta}\} \cdot \sum_{i=1}^{n_j} (\Lambda_{0\gamma}(t_{ij}) + \Lambda_0(t_{ij}) X_{ij\gamma}) \exp\{\mathbf{X}_{ij}^T \boldsymbol{\beta}\}, \quad \alpha, \gamma = 1:p
\end{aligned}
$$

$$
\begin{aligned}
\frac{\partial^2 l_2^j}{\partial \pi_g \partial w_q} = & \mathbf{1}_{\{q=g\}} \left\{ \frac{w_g^{D_j-1} \exp\{-w_g \sum_{i=1}^{n_j} \Lambda_0(t_{ij}) \exp\left(\mathbf{X}_{ij}^T \boldsymbol{\beta}\right)\} \left(D_j - w_g \sum_{i=1}^{n_j} \Lambda_0(t_{ij}) \exp\left(\mathbf{X}_{ij}^T \boldsymbol{\beta}\right)\right)}{\sum_{k=1}^K \pi_k w_k^{D_j} \cdot \exp\{-w_k \sum_{i=1}^{n_j} \Lambda_0(t_{ij}) \exp\left(\mathbf{X}_{ij}^T \boldsymbol{\beta}\right)\}} \right\} + \\
& + \mathbf{1}_{\{q=K\}} \left\{ - \frac{w_K^{D_j-1} \exp\{-w_K \sum_{i=1}^{n_j} \Lambda_0(t_{ij}) \exp\left(\mathbf{X}_{ij}^T \boldsymbol{\beta}\right)\} \left(D_j - w_K \sum_{i=1}^{n_j} \Lambda_0(t_{ij}) \exp\left(\mathbf{X}_{ij}^T \boldsymbol{\beta}\right)\right)}{\sum_{k=1}^K \pi_k w_k^{D_j} \cdot \exp\{-w_k \sum_{i=1}^{n_j} \Lambda_0(t_{ij}) \exp\left(\mathbf{X}_{ij}^T \boldsymbol{\beta}\right)\}} \right\} + \\
& - \frac{\left(w_g^{D_j} \exp\left\{-w_g \sum_{i=1}^{n_j} \Lambda_0(t_{ij}) \exp\left(\mathbf{X}_{ij}^T \boldsymbol{\beta}\right)\right\} - w_K^{D_j} \exp\left\{-w_K \sum_{i=1}^{n_j} \Lambda_0(t_{ij}) \exp\left(\mathbf{X}_{ij}^T \boldsymbol{\beta}\right)\right\}\right)}{\left(\sum_{k=1}^K \pi_k w_k^{D_j} \cdot \exp \sum_{i=1}^{n_j} \left[-\Lambda_0(t_{ij}) w_k \exp\left(\mathbf{X}_{ij}^T \boldsymbol{\beta}\right)\right]\right)^2} \cdot \\
& \cdot \left(\pi_q w_q^{D_j-1} \exp\{-w_q \sum_{i=1}^{n_j} \Lambda_0(t_{ij}) \exp\left(\mathbf{X}_{ij}^T \boldsymbol{\beta}\right)\} \left(D_j - w_q \sum_{i=1}^{n_j} \Lambda_0(t_{ij}) \exp\left(\mathbf{X}_{ij}^T \boldsymbol{\beta}\right)\right)\right), \quad g = 1:(K-1), q = 1:K
\end{aligned}
$$

$$
\begin{aligned}
\frac{\partial^2 l_2^j}{\partial \pi_g \partial \boldsymbol{\beta}_\alpha} = & \sum_{i=1}^{n_j} (\Lambda_{0\alpha}(t_{ij}) + \Lambda_0(t_{ij}) X_{ij\alpha}) \exp\{\mathbf{X}_{ij}^T \boldsymbol{\beta}\} \cdot \\
& \Bigg\{ \frac{w_K^{D_j+1} \exp\{-w_K \sum_{i=1}^{n_j} \Lambda_0(t_{ij}) \exp\left(\mathbf{X}_{ij}^T \boldsymbol{\beta}\right)\} - w_g^{D_j+1} \exp\{-w_g \sum_{i=1}^{n_j} \Lambda_0(t_{ij}) \exp\left(\mathbf{X}_{ij}^T \boldsymbol{\beta}\right)\}}{\sum_{k=1}^K \pi_k w_k^{D_j} \cdot \exp\{-w_k \sum_{i=1}^{n_j} \Lambda_0(t_{ij}) \exp\left(\mathbf{X}_{ij}^T \boldsymbol{\beta}\right)\}} + \\
& - \frac{w_K^{D_j} \exp\{-w_K \sum_{i=1}^{n_j} \Lambda_0(t_{ij}) \exp\left(\mathbf{X}_{ij}^T \boldsymbol{\beta}\right)\} - w_g^{D_j} \exp\{-w_g \sum_{i=1}^{n_j} \Lambda_0(t_{ij}) \exp\left(\mathbf{X}_{ij}^T \boldsymbol{\beta}\right)\}}{(\sum_{k=1}^K \pi_k w_k^{D_j} \cdot \exp\{-w_k \sum_{i=1}^{n_j} \Lambda_0(t_{ij}) \exp\left(\mathbf{X}_{ij}^T \boldsymbol{\beta}\right)\})^2} \\
& \cdot \sum_{k=1}^K \pi_k w_k^{D_j+1} \cdot \exp\{-w_k \sum_{i=1}^{n_j} \Lambda_0(t_{ij}) \exp\left(\mathbf{X}_{ij}^T \boldsymbol{\beta}\right)\} \Bigg\}, \quad g = 1:(K-1), \alpha = 1:p
\end{aligned}
$$

$$
\begin{aligned}
\frac{\partial^2 l_2^j}{\partial w_q \partial \boldsymbol{\beta}_\alpha} = & \pi_q w_q^{D_j-1} \exp\{-w_g \sum_{i=1}^{n_j} \Lambda_0(t_{ij}) \exp\left(\mathbf{X}_{ij}^T \boldsymbol{\beta}\right)\} \sum_{i=1}^{n_j} (\Lambda_{0\alpha}(t_{ij}) + \Lambda_0(t_{ij}) X_{ij\alpha}) \exp\{\mathbf{X}_{ij}^T \boldsymbol{\beta}\} \cdot \\
& \Bigg\{ \frac{-w_q(D_j + 1 - w_q \sum_{i=1}^{n_j} \Lambda_0(t_{ij}) \exp\left(\mathbf{X}_{ij}^T \boldsymbol{\beta}\right))}{\sum_{k=1}^K \pi_k w_k^{D_j} \cdot \exp\{-w_k \sum_{i=1}^{n_j} \Lambda_0(t_{ij}) \exp\left(\mathbf{X}_{ij}^T \boldsymbol{\beta}\right)\}} + \\
& + \frac{(D_j - w_q \sum_{i=1}^{n_j} \Lambda_0(t_{ij}) \exp\left(\mathbf{X}_{ij}^T \boldsymbol{\beta}\right)) \sum_{k=1}^K \pi_k w_k^{D_j+1} \cdot \exp\{-w_k \sum_{i=1}^{n_j} \Lambda_0(t_{ij}) \exp\left(\mathbf{X}_{ij}^T \boldsymbol{\beta}\right)\}}{(\sum_{k=1}^K \pi_k w_k^{D_j} \cdot \exp\{-w_k \sum_{i=1}^{n_j} \Lambda_0(t_{ij}) \exp\left(\mathbf{X}_{ij}^T \boldsymbol{\beta}\right)\})^2} \Bigg\}, \quad q = 1:K, \alpha = 1:p
\end{aligned}
$$

Louis method

We start from the jth component of the full loglikelihood, written in Eq. (A.2).

$$l^j_{full}(\boldsymbol{\theta}; data|\mathbf{z}) = \sum_{k=1}^{K} z_{jk} \cdot \left[\log(\pi_k) + \left\{ \sum_{i=1}^{n_j} \delta_{ij}[\log(\lambda_0(t_{ij})) + \log(w_k) + \mathbf{X}_{ij}^T \boldsymbol{\beta}] - \Lambda_0(t_{ij}) w_k \exp\{\mathbf{X}_{ij}^T \boldsymbol{\beta}\} \right\} \right]. \tag{A.2}$$

Louis states that the jth component of the observed information matrix $\mathbf{I}$ can be written as follows:

$$\mathbf{I}^j = \mathbf{E}[B_j(T_{ij}, \delta_{ij}, z_{jk})] - \mathbf{E}[S_j(T_{ij}, \delta_{ij}, z_{jk}) S_j(T_{ij}, \delta_{ij}, z_{jk})^T] + S_j(T_{ij}, \delta_{ij})^\star S_j(T_{ij}, \delta_{ij})^{\star T}. \tag{A.3}$$

The final observed information matrix is the sum of all j^{th} elements: $\mathbf{I} = \sum_{j=1}^{J} \mathbf{I}^j$.

We define S and $S^\star$ as the gradient vectors of the full loglikelihood and the observable loglikelihood, see (A.4) and (A.5), while B and $B^\star$ are the negative second derivative matrices of full loglikelihood and the observable loglikelihood, see (A.7). We remind that the unique observable variables are (T_{ij}, δ_{ij}), while the complete set of variables is $(T_{ij}, \delta_{ij}, z_{jk})$.

$$S_j^T(T_{ij}, \delta_{ij}, z_{jk}) = \nabla l^j_{full} = \left[\frac{\partial l^j_{full}}{\partial \pi_1}, \dots, \frac{\partial l^j_{full}}{\partial \pi_{K-1}}, \frac{\partial l^j_{full}}{\partial w_1}, \dots, \frac{\partial l^j_{full}}{\partial w_K}, \frac{\partial l^j_{full}}{\partial \boldsymbol{\beta}_1}, \dots, \frac{\partial l^j_{full}}{\partial \boldsymbol{\beta}_p} \right]^T \tag{A.4}$$

$$S_j^\star(T_{ij}, \delta_{ij}) = \mathbf{E}[S_j(T_{ij}, \delta_{ij}, z_{jk})] \tag{A.5}$$

$$\mathbf{E}[S_j(T_{ij}, \delta_{ij}, z_{jk}) S_j(T_{ij}, \delta_{ij}, z_{jk})^T] = \left[\begin{array}{c|c|c} \mathbf{E}[\nabla_\pi l^j_{full} \nabla_\pi^T l^j_{full}] & \mathbf{E}[\nabla_\pi l^j_{full} \nabla_w^T l^j_{full}] & \mathbf{E}[\nabla_\pi l^j_{full} \nabla_{\boldsymbol{\beta}}^T l^j_{full}] \\ \hline \mathbf{E}[\nabla_w l^j_{full} \nabla_\pi^T l^j_{full}] & \mathbf{E}[\nabla_w l^j_{full} \nabla_w^T l^j_{full}] & \mathbf{E}[\nabla_w l^j_{full} \nabla_{\boldsymbol{\beta}}^T l^j_{full}] \\ \hline \mathbf{E}[\nabla_{\boldsymbol{\beta}} l^j_{full} \nabla_\pi^T l^j_{full}] & \mathbf{E}[\nabla_{\boldsymbol{\beta}} l^j_{full} \nabla_w^T l^j_{full}] & \mathbf{E}[\nabla_{\boldsymbol{\beta}} l^j_{full} \nabla_{\boldsymbol{\beta}}^T l^j_{full}] \end{array} \right] \tag{A.6}$$

$$B_j(T_{ij}, \delta_{ij}, z_{jk}) =$$
$$\begin{pmatrix} -\frac{\partial^2 l^j_{full}}{\partial \pi_1^2} & \cdots & -\frac{\partial^2 l^j_{full}}{\partial \pi_1 \partial \pi_{K-1}} & & & & & & \\ \vdots & \ddots & \vdots & & & & & & \\ -\frac{\partial^2 l^j_{full}}{\partial \pi_{K-1} \partial \pi_1} & \cdots & -\frac{\partial^2 l^j_{full}}{\partial \pi_{K-1}^2} & & & & & & \\ & & & -\frac{\partial^2 l^j_{full}}{\partial w_1^2} & & & -\frac{\partial^2 l^j_{full}}{\partial w_1 \partial \boldsymbol{\beta}_1} & \cdots & -\frac{\partial^2 l^j_{full}}{\partial w_1 \partial \boldsymbol{\beta}_p} \\ & & & & \ddots & & \vdots & \vdots & \vdots \\ & & & & & -\frac{\partial^2 l^j_{full}}{\partial w_K^2} & -\frac{\partial^2 l^j_{full}}{\partial w_K \partial \boldsymbol{\beta}_1} & \cdots & -\frac{\partial^2 l^j_{full}}{\partial w_K \partial \boldsymbol{\beta}_p} \\ & & & -\frac{\partial^2 l^j_{full}}{\partial \boldsymbol{\beta}_1 \partial w_1} & \cdots & -\frac{\partial^2 l^j_{full}}{\partial \boldsymbol{\beta}_1 \partial w_k} & -\frac{\partial^2 l^j_{full}}{\partial \boldsymbol{\beta}_1^2} & \cdots & -\frac{\partial^2 l^j_{full}}{\partial \boldsymbol{\beta}_1 \partial \boldsymbol{\beta}_p} \\ & & & \vdots & \vdots & \vdots & \vdots & \ddots & \vdots \\ & & & -\frac{\partial^2 l^j_{full}}{\partial \boldsymbol{\beta}_p \partial w_1} & \cdots & -\frac{\partial^2 l^j_{full}}{\partial \boldsymbol{\beta}_p \partial w_K} & -\frac{\partial^2 l^j_{full}}{\partial \boldsymbol{\beta}_p \partial \boldsymbol{\beta}_1} & \cdots & -\frac{\partial^2 l^j_{full}}{\partial \boldsymbol{\beta}_p^2} \end{pmatrix} \tag{A.7}$$

The components of S are computed in (A.8), (A.9) and (A.10).

$$\frac{\partial l^j_{full}}{\partial \pi_g} = \frac{z_{jg}}{\pi_g} - \frac{z_{jK}}{\pi_K}, \qquad g = 1:(K-1) \tag{A.8}$$

$$\frac{\partial l^j_{full}}{\partial w_q} = z_{jq} \sum_{i=1}^{n_j} \frac{\delta_{ij}}{w_q} - \Lambda_0(t_{ij}) \exp\left\{\mathbf{X}^T_{ij}\boldsymbol{\beta}\right\}, \quad q = 1:K \tag{A.9}$$

$$\frac{\partial l^j_{full}}{\partial \boldsymbol{\beta}_\alpha} = \sum_{k=1}^{K} z_{jk} \sum_{i=1}^{n_j} \delta_{ij} \left\{ \frac{\lambda_{0\alpha}(t_{ij})}{\lambda_0(t_{ij})} + X^T_{ij\alpha} \right\} - w_k \exp\left\{\mathbf{X}^T_{ij}\boldsymbol{\beta}\right\} \left\{\Lambda_{0\alpha}(t_{ij}) + \Lambda_0(t_{ij}) X_{ij\alpha}\right\}, \quad \alpha = 1:p \tag{A.10}$$

The components of $\mathbf{E}[S_j(T_{ij}, \delta_{ij}, z_{jk}) S_j(T_{ij}, \delta_{ij}, z_{jk})^T]$ are:

$$\mathbf{E}\left[\frac{\partial l^j_{full}}{\partial \pi_g}\frac{\partial l^j_{full}}{\partial \pi_l}\right] = \frac{\alpha_{jg}}{\pi^2_g}\mathbf{1}_{\{g=l\}} + \frac{\alpha_{jK}}{\pi^2_K}, \qquad g, l = 1:(K-1) \tag{A.11}$$

$$\mathbf{E}\left[\frac{\partial l^j_{full}}{\partial w_q}\frac{\partial l^j_{full}}{\partial w_r}\right] = \mathbf{1}_{\{q=r\}} \cdot \alpha_{jq} \left(\sum_{i=1}^{n_j} \frac{\delta_{ij}}{w_q} - \Lambda_0(t_{ij}) \exp\left\{\mathbf{X}^T_{ij}\boldsymbol{\beta}\right\} \right)^2, \qquad q, r = 1:K \tag{A.12}$$

$$\begin{aligned}\mathbf{E}\left[\frac{\partial l^j_{full}}{\partial \boldsymbol{\beta}_\alpha}\frac{\partial l^j_{full}}{\partial \boldsymbol{\beta}_\gamma}\right] =& \sum_{k=1}^{K} \alpha_{jk} \left(\sum_{i=1}^{n_j} \delta_{ij} \left\{ \frac{\lambda_{0\alpha}(t_{ij})}{\lambda_0(t_{ij})} + X^T_{ij\alpha} \right\} - w_k \exp\left\{\mathbf{X}^T_{ij}\boldsymbol{\beta}\right\} \left\{\Lambda_{0\alpha}(t_{ij}) + \Lambda_0(t_{ij}) X_{ij\alpha}\right\} \right) \cdot \\ & \left(\sum_{i=1}^{n_j} \delta_{ij} \left\{ \frac{\lambda_{0\gamma}(t_{ij})}{\lambda_0(t_{ij})} + X^T_{ij\gamma} \right\} - w_k \exp\left\{\mathbf{X}^T_{ij}\boldsymbol{\beta}\right\} \left\{\Lambda_{0\gamma}(t_{ij}) + \Lambda_0(t_{ij}) X_{ij\gamma}\right\} \right), \qquad \alpha, \gamma = 1:p\end{aligned} \tag{A.13}$$

$$\mathbf{E}\left[\frac{\partial l^j_{full}}{\partial \pi_g}\frac{\partial l^j_{full}}{\partial w_q}\right] = \begin{cases} \frac{\alpha_{jg}}{\pi_g} \sum_{i=1}^{n_j} \frac{\delta_{ij}}{w_g} - \Lambda_0(t_{ij}) \exp\left\{\mathbf{X}^T_{ij}\boldsymbol{\beta}\right\}, \text{if} \quad q = g \\ -\frac{\alpha_{jK}}{\pi_K} \sum_{i=1}^{n_j} \frac{\delta_{ij}}{w_K} - \Lambda_0(t_{ij}) \exp\left\{\mathbf{X}^T_{ij}\boldsymbol{\beta}\right\}, \text{if} \quad q = K \end{cases} \tag{A.14}$$

$$\begin{aligned}\mathbf{E}\left[\frac{\partial l^j_{full}}{\partial \pi_g}\frac{\partial l^j_{full}}{\partial \boldsymbol{\beta}_\alpha}\right] =& \frac{\alpha_{jg}}{\pi_g} \sum_{i=1}^{n_j} \delta_{ij} \left\{ \frac{\lambda_{0\alpha}(t_{ij})}{\lambda_0(t_{ij})} + X^T_{ij\alpha} \right\} - w_g \exp\left\{\mathbf{X}^T_{ij}\boldsymbol{\beta}\right\} \left\{\Lambda_{0\alpha}(t_{ij}) + \Lambda_0(t_{ij}) X_{ij\alpha}\right\} \\ & - \frac{\alpha_{jK}}{\pi_K} \sum_{i=1}^{n_j} \delta_{ij} \left\{ \frac{\lambda_{0\alpha}(t_{ij})}{\lambda_0(t_{ij})} + X^T_{ij\alpha} \right\} - w_K \exp\left\{\mathbf{X}^T_{ij}\boldsymbol{\beta}\right\} \left\{\Lambda_{0\alpha}(t_{ij}) + \Lambda_0(t_{ij}) X_{ij\alpha}\right\}, \quad g = 1:(K-1), \alpha = 1:p\end{aligned} \tag{A.15}$$

$$\mathbf{E}\left[\frac{\partial l^j_{full}}{\partial w_q}\frac{\partial l^j_{full}}{\partial \boldsymbol{\beta}_\alpha}\right] = \alpha_{jg} \sum_{i=1}^{n_j} \delta_{ij} \left\{ \frac{\lambda_{0\alpha}(t_{ij})}{\lambda_0(t_{ij})} + X^T_{ij\alpha} \right\} - w_q \exp\left\{\mathbf{X}^T_{ij}\boldsymbol{\beta}\right\} \left\{\Lambda_{0\alpha}(t_{ij}) + \Lambda_0(t_{ij}) X_{ij\alpha}\right\} \cdot \sum_{i=1}^{n_j} \frac{\delta_{ij}}{w_q} - \Lambda_0(t_{ij}) \exp\left\{\mathbf{X}^T_{ij}\boldsymbol{\beta}\right\} \tag{A.16}$$

The components of B are computed in (A.17), (A.18), (A.19) and (A.20).

$$\frac{\partial^2 l^j_{full}}{\partial \pi_g \partial \pi_l} = -\frac{z_{jg}}{\pi_g^2}\mathbf{1}_{\{g=l\}} - \frac{z_{jK}}{\pi_K^2} \qquad g, l \in \{1, .., K-1\} \tag{A.17}$$

$$\frac{\partial^2 l^j_{full}}{\partial w_q^2} = -\, z_{jq} \sum_{i=1}^{n_j} \frac{\delta_{ij}}{w_q^2} \quad q \in \{1, .., K\} \tag{A.18}$$

$$\begin{aligned}\frac{\partial^2 l^j_{full}}{\partial \boldsymbol{\beta}_\alpha \partial \boldsymbol{\beta}_\gamma} =& \sum_{k=1}^{K} z_{jk} \sum_{i=1}^{n_j} \delta_{ij} \left\{ \frac{\lambda_{0\alpha\gamma}(t_{ij})\lambda_0(t_{ij}) - \lambda_{0\alpha}(t_{ij})\lambda_{0\gamma}(t_{ij})}{\lambda_0(t_{ij})^2} \right\} - \\ & w_k \exp\left\{\mathbf{X}_{ij}^T \boldsymbol{\beta}\right\} \{\Lambda_{0\alpha\gamma}(t_{ij}) + \Lambda_{0\alpha}(t_{ij})X_{ij\gamma} + \Lambda_{0\gamma}(t_{ij})X_{ij\alpha} + \Lambda_0(t_{ij})X_{ij\alpha}X_{ij\gamma}\} \quad \alpha, \gamma = 1:p\end{aligned} \tag{A.19}$$

$$\frac{\partial^2 l^j_{full}}{\partial w_q \partial \boldsymbol{\beta}_\alpha} = z_{jq} \sum_{i=1}^{n_j} - \left(\Lambda_0(t_{ij})X_{ij\alpha} + \Lambda_{0\alpha}(t_{ij})\right) \exp\left\{\mathbf{X}_{ij}^T \boldsymbol{\beta}\right\} \quad q = 1:K, \alpha = 1:p \tag{A.20}$$

B. Simulation case study (i): fixed K and frailty ratio, higher group size

In Appendix B we show the results of the first simulation study with $K = 2$ hidden populations, a constant frailty ratio of $w_2/w_1 = 1.55$ and the proportion π_1 belonging to the first population varied in 9 scenarios (Table B.1). In this case, we consider 50 statistical units per group.

	π_1	w_1	w_2	ratio
1	0.1	0.8	1.24	1.55
2	0.2	0.8	1.24	1.55
3	0.3	0.8	1.24	1.55
4	0.4	0.8	1.24	1.55
5	0.5	0.8	1.24	1.55
6	0.6	0.8	1.24	1.55
7	0.7	0.8	1.24	1.55
8	0.8	0.8	1.24	1.55
9	0.9	0.8	1.24	1.55

Table B.1. First simulation study

In Table B.2, we present the resulting estimates of K, the number of latent populations, using three alternative methods of model selection. Each column represents one of the nine scenarios with alternative values of π_1, while each block is related to a selection criterion. The majority of simulations estimate the correct value of $K = 2$ for all three model selection methods, otherwise for AIC and BIC $K = 1$ is the next most common estimate, and, for the method of Laird (1978) $K = 3$. The symmetric pattern of the table illustrates that using AIC or BIC, the correct $K = 2$ is estimated more frequently when the mixing proportion π_1 is closer to 0.5.

Figure B.1 shows that the mixing proportion π_1 is well estimated in all scenarios. Figure B.2 shows that the frailty ratio of 1.55 tends to be estimated more accurately when the mixing proportion is closer to 0.5. The case characterised by the

	Population	0.1	0.2	0.3	0.4	0.5	0.6	0.7	0.8	0.9
	1	136	22	3	0	1	4	12	74	362
	2	858	971	991	997	996	994	985	923	636
**AIC**	3	6	7	6	3	3	2	3	3	2
	4	0	0	0	0	0	0	0	0	0
	5	0	0	0	0	0	0	0	0	0
	1	285	57	12	4	6	11	39	190	642
	2	715	943	988	996	994	989	961	810	358
**BIC**	3	0	0	0	0	0	0	0	0	0
	4	0	0	0	0	0	0	0	0	0
	5	0	0	0	0	0	0	0	0	0
	1	0	0	0	0	0	0	0	0	15
	2	677	620	421	577	683	747	785	810	836
**Laird**	3	295	339	542	383	269	206	175	154	98
	4	28	41	31	39	43	46	39	35	50
	5	0	0	6	1	5	1	1	1	1

Table B.2. Estimates of number of latent populations, K, in first simulation study (fixed frailty ratio and nine alternative values of π_1). The table is split in three main blocks according to the criterium used for the estimates. From the top to the bottom we have: AIC, BIC and Laird's criterion (Laird, 1978). Each column in each block sums to 1000 (the total number of simulations per case). The green bars highlight the total number of simulations in which the best model estimates $K = 2$, which is also the true model.

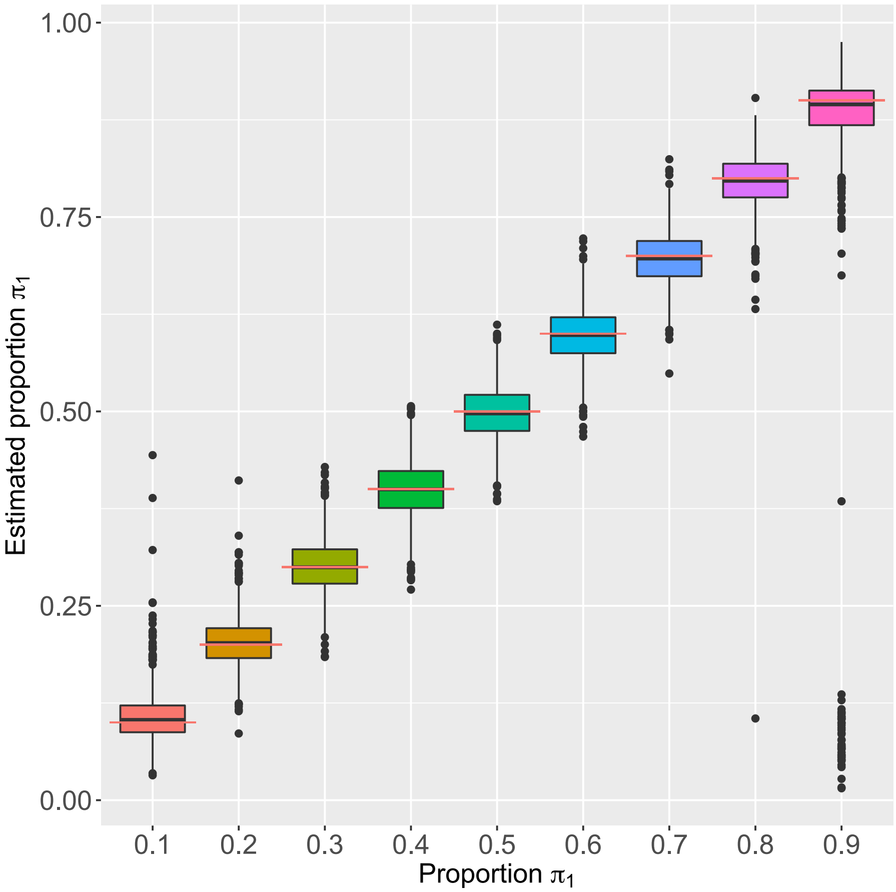

Fig. B.1. Estimates of π_1, fixed frailty ratio and nine alternative values of π_1. We show the boxplots (median and quantiles) of the maximum likelihood estimators for π_1 over all 1000 simulations, for each of nine cases. The red lines represent the real values.

lowest performance of the algorithm is $\pi_1 = 0.9$. Indeed two populations are detected in 636 datasets out of 1,000 according to AIC (Table B.2) and several outliers are detected in both Figure B.1 and B.2.

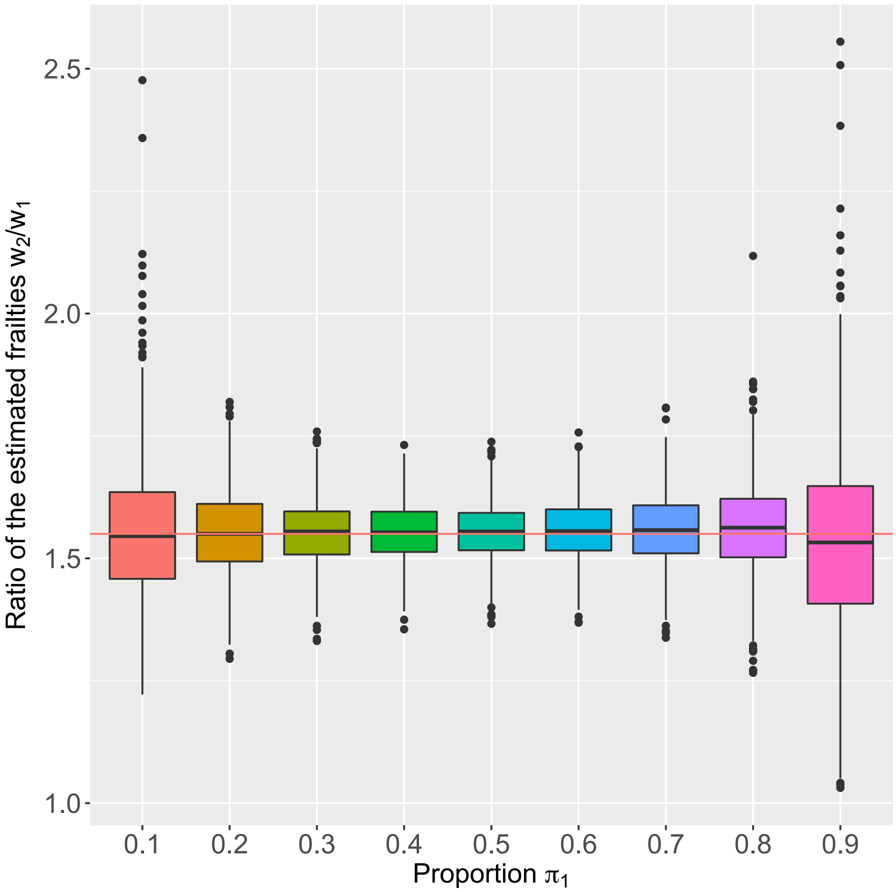

Fig. B.2. Estimates of the ratio w_2/w_1, fixed frailty ratio and nine alternative values of π_1. We show the boxplots (median and quantiles) of the ratio of the maximum likelihood estimators for w_1 and w_2 over all 1000 simulations, for each of nine cases. The red lines represent the real values.

C. Simulation case study (ii): fixed K and frailty ratio, lower group size

In this case, we propose the same scenarios of Table B.1, considering 35 statistical units per group. It is immediate to notice that Figure C.1 and Figure C.2 are almost identical to Figure B.1 and Figure B.2, respectively. This means that the estimates of the ratio and of the mixing proportions are robust with respect to group size. On the other hand, by comparing Table C.1 and Table B.2 we notice a lower performance for both AIC and BIC in detecting the right number of latent populations. The method of Laird (1978) seems to be robust and consistent with respect to group size.

D. Simulation case study (iii): fixed K and $\boldsymbol{\pi}$

In Appendix D we show the results of the third simulation study with $K = 2$, the proportion of groups with the lower frailty value fixed at $\pi_1 = 0.3$, and the frailty ratio w_2/w_1 varying in 7 scenarios from 1.14 to 3, see Table D.1. We have 50 statistical units per group in each scenario.

As before, in Table D.2, we present the estimates of K, the number of latent populations. Broadly for all three model selection methods, the larger the contrast in frailties w_2/w_1 between the populations, the more frequently the true $K = 2$ is

	Population	0.1	0.2	0.3	0.4	0.5	0.6	0.7	0.8	0.9
	1	567	372	262	223	217	279	418	592	789
	2	430	624	731	773	779	717	576	407	209
**AIC**	3	3	4	7	4	4	4	6	1	2
	4	0	0	0	0	0	0	0	0	0
	5	0	0	0	0	0	0	0	0	0
	1	777	556	424	391	401	485	621	802	938
	2	223	444	575	609	599	515	379	198	62
**BIC**	3	0	0	1	0	0	0	0	0	0
	4	0	0	0	0	0	0	0	0	0
	5	0	0	0	0	0	0	0	0	0
	1	0	0	0	0	0	0	0	0	52
	2	669	588	476	566	674	729	729	768	758
**Laird**	3	292	372	482	399	281	222	236	199	137
	4	38	37	38	28	42	47	33	33	52
	5	1	3	4	7	3	2	2	0	1

Table C.1. Estimates of number of latent populations, K, in first simulation study (fixed frailty ratio and nine alternative values of π_1). The table is split in three main blocks according to the criterium used for the estimates. From the top to the bottom we have: AIC, BIC and Laird's criterion (Laird, 1978). Each column in each block sums to 1000 (the total number of simulations per case). The green bars highlight the total number of simulations in which the best model estimates $K = 2$, which is also the true model.

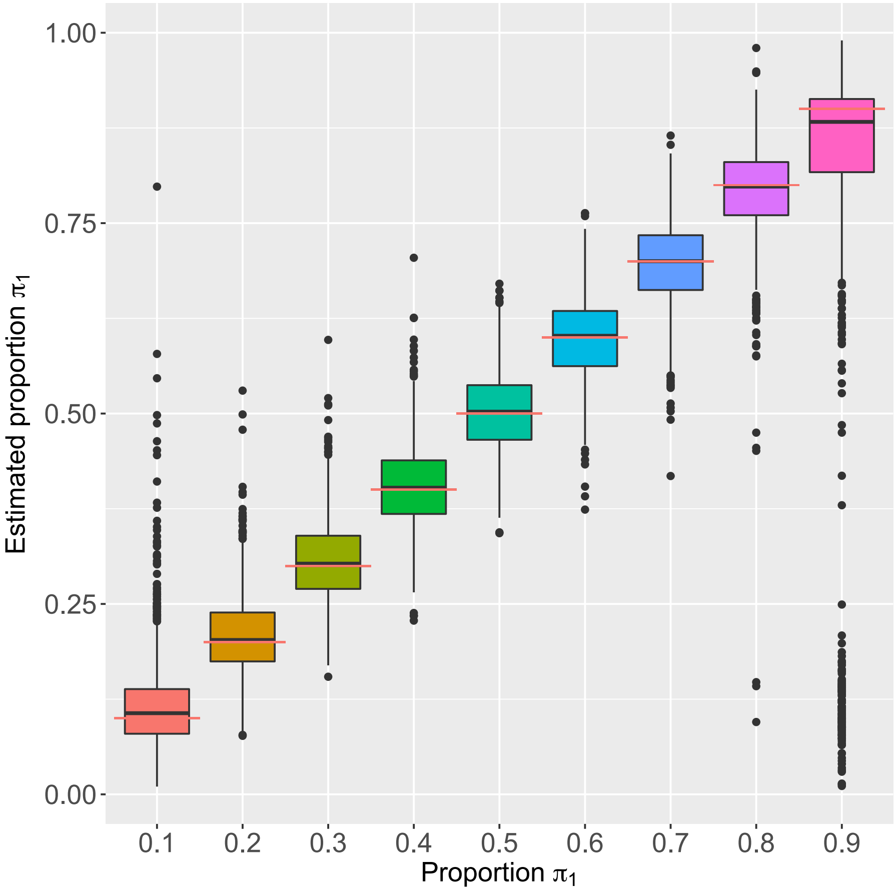

Fig. C.1. Estimates of π_1, fixed frailty ratio and nine alternative values of π_1. We show the boxplots (median and quantiles) of the maximum likelihood estimators for π_1 over all 1000 simulations, for each of nine cases. The red lines represent the real values.

obtained.

The estimates of the mixing proportion are represented in Figure D.1, and the estimates of the frailty ratio in Figure D.2, showing that more accurate estimates are obtained as the frailty ratio increases, thus as the contrast between the two

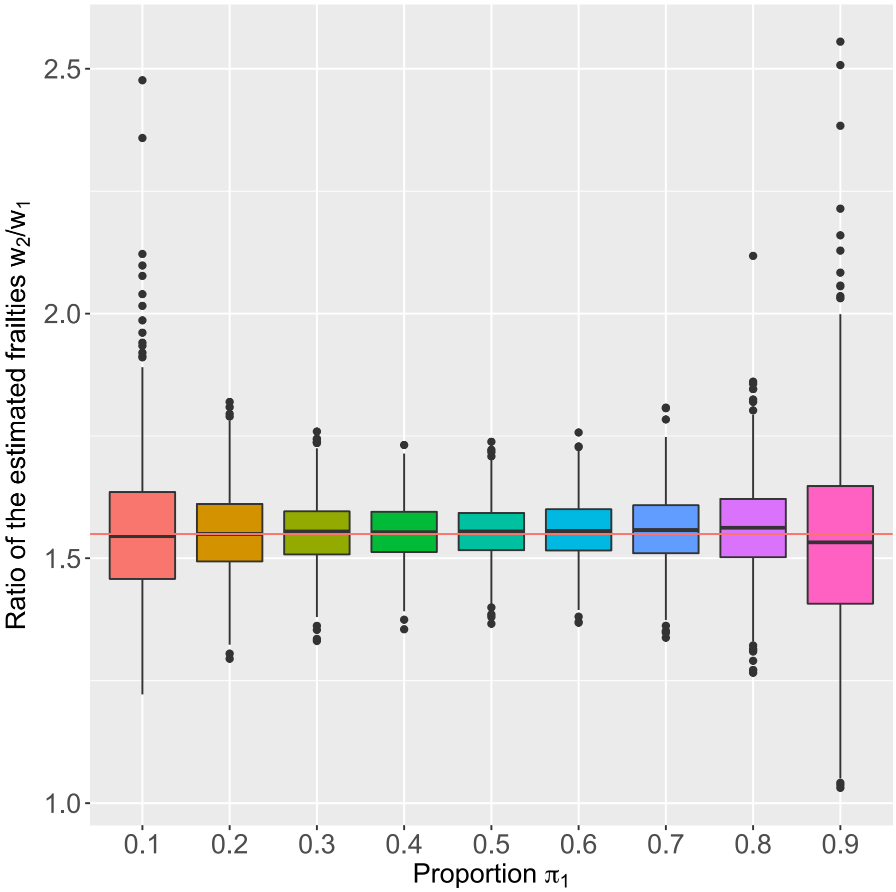

Fig. C.2. Estimates of the ratio w_2/w_1, fixed frailty ratio and nine alternative values of π_1. We show the boxplots (median and quantiles) of the ratio of the maximum likelihood estimators for w_1 and w_2 over all 1000 simulations, for each of nine cases. The red lines represent the real values.

	π_1	w_1	w_2	ratio
1	0.3	0.7	0.8	1.14
2	0.3	0.7	0.9	1.29
3	0.3	0.7	1	1.43
4	0.3	0.7	1.1	1.57
5	0.3	0.7	1.2	1.71
6	0.3	0.7	1.4	2
7	0.3	0.7	2.1	3

Table D.1. Third simulation study

populations becomes greater.

E. Simulation case study (iv): evaluating K estimate

In Appendix E, we show the full results of the fourth simulation study, where the number of latent populations is varied in three scenarios with $K = \{1, 2, 3, 4\}$ in turn, while the mixing proportions and frailty ratios are fixed at values listed in Table E.1.

In Table E.2, we present the estimates of K, the number of latent populations, under three methods of model selection. We note that AIC estimates the true value of K in the majority of simulations in the three considered frameworks, top block

	Population	1.14	1.29	1.43	1.57	1.71	2	3
	1	994	984	337	0	0	0	0
	2	6	16	659	995	997	998	992
**AIC**	3	0	0	4	5	3	2	8
	4	0	0	0	0	0	0	0
	5	0	0	0	0	0	0	0
	1	1000	997	547	4	0	0	0
	2	0	3	453	996	1000	1000	1000
**BIC**	3	0	0	0	0	0	0	0
	4	0	0	0	0	0	0	0
	5	0	0	0	0	0	0	0
	1	274	2	0	0	0	0	0
	2	622	672	541	417	436	542	651
**Laird**	3	92	301	432	542	512	381	235
	4	12	25	26	33	46	72	102
	5	0	0	1	8	6	5	12

Table D.2. Estimates of number of latent populations, K, in second simulation study (fixed π_1 and 7 alternative values of w_2/w_1). The table is split in three main blocks according to the criterium used for the estimates. From the top to the bottom we have: AIC, BIC and Laird's criterion (Laird, 1978). Each column in each block sums to 1000 (the total number of simulations per case). The green bars highlight the total number of simulations in which the best model estimates $K = 2$, which is also the true model.

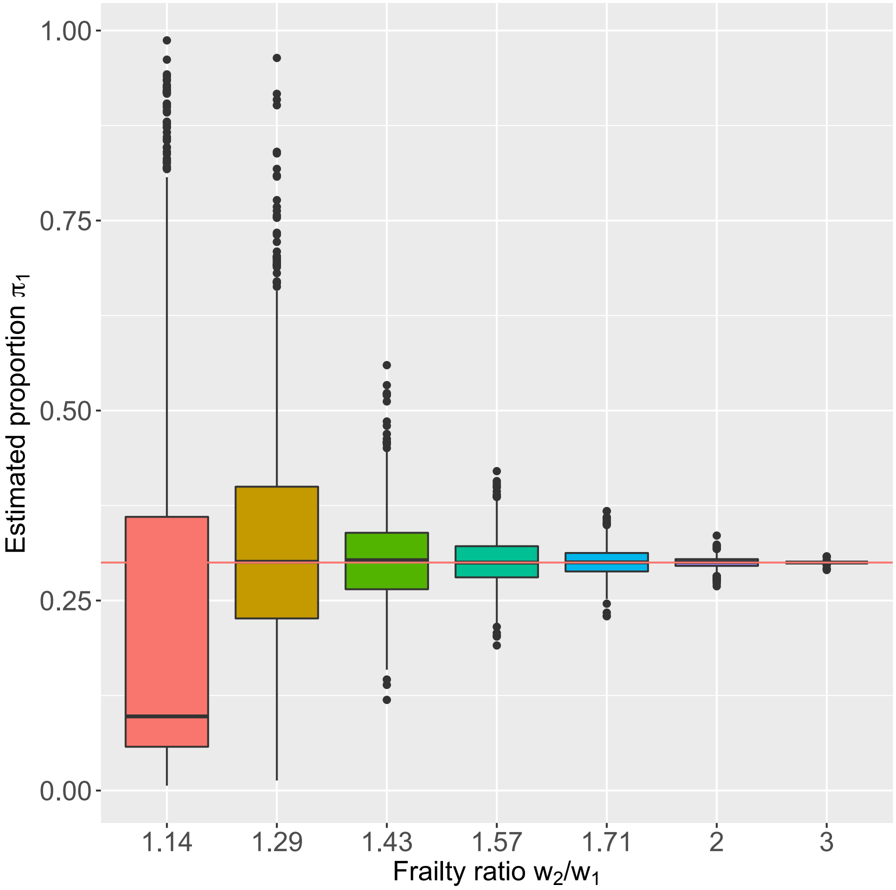

Fig. D.1. Estimates of π_1, fixed π_1 and seven alternative values of w_2/w_1. We represent the boxplots (median and quantiles) of the maximum likelihood estimators for π_1 over all 1000 simulations, for each of the seven cases. The red lines represent the real values.

of Table E.2. Both AIC and BIC show the best performances in the case of real $K = 2$ and the worst performances in the case of real $K = 4$. BIC estimates one latent population less than the true value in the majority of cases for both real $K = 3$ and $K = 4$, central block of Table E.2. Finally, the method proposed by Laird (1978) tends to estimate the true K in about

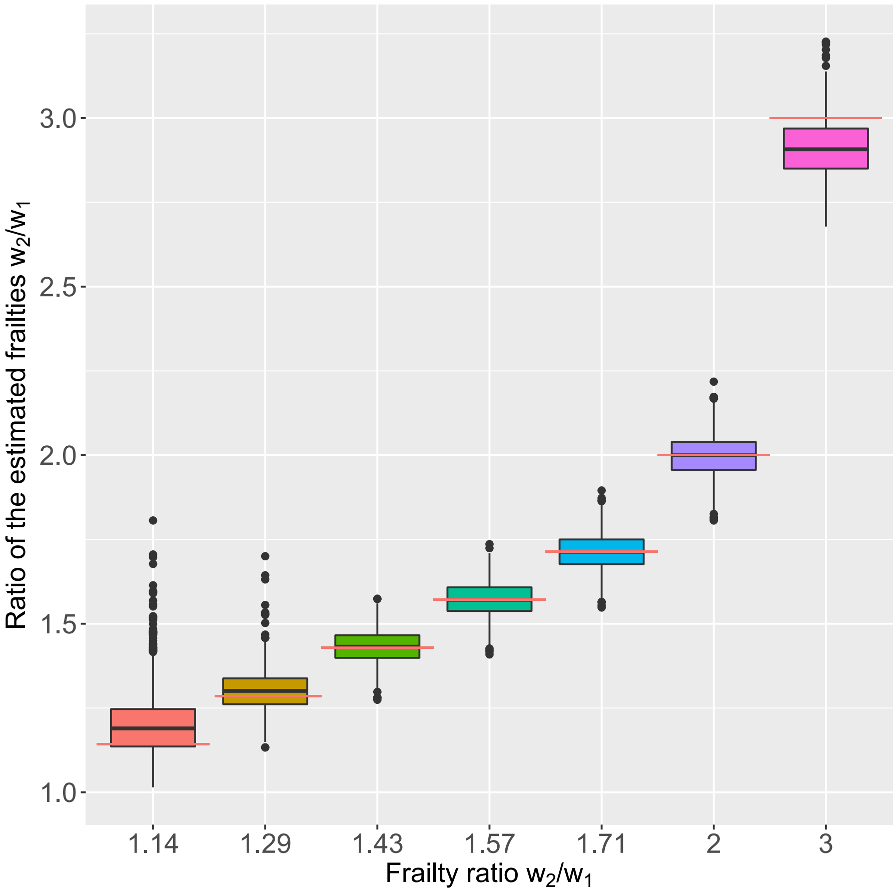

Fig. D.2. Estimates of w_2/w_1, fixed π_1 and seven alternative values of w_2/w_1. We represent the boxplots (median and quantiles) of the ratio of the maximum likelihood estimators for w_2 and w_1 over all 1000 simulations, for each of the seven cases. The red lines represent the real values.

K	π_1	π_2	π_3	π_4	w_1	w_2	w_3	w_4	ratio
1	1	-	-	-	1	-	-	-	-
2	0.40	0.60	-	-	2	3	-	-	1.5
3	0.20	0.30	0.50	-	2	3	5	-	[1.5,2.5]
4	0.15	0.25	0.30	0.30	2	3	5	8	[1.5,2.5,4]

Table E.1. Fourth simulation study

half of the simulations, but in the other half it tends to estimate one or two latent populations more than the true value, last block from the top of Table E.2.

The mixing proportions (Figure E.1) and the frailty ratios (Figure E.2) are estimated accurately.

References

Laird, N. (1978). Nonparametric maximum likelihood estimation of a mixing distribution. *Journal of the American Statistical Association* **73**(364), 805–811.

[*Received August 1, 2010; revised October 1, 2010; accepted for publication November 1, 2010*]

	Population	1	2	3	4
	1	1000	29	0	0
	2	0	968	260	0
**AIC**	3	0	3	739	320
	4	0	0	1	680
	5	0	0	0	0
	1	1000	65	0	0
	2	0	935	524	5
**BIC**	3	0	0	476	609
	4	0	0	0	386
	5	0	0	0	0
	1	734	0	0	0
	2	243	590	0	0
**Laird**	3	19	377	483	0
	4	4	31	409	497
	5	0	2	108	503

Table E.2. Estimates of number of latent populations, K, in third simulation study. In this case, we vary all the parameters up to Table E.1. The table is split in three main blocks according to the criterium used for the estimates. From the top to the bottom we have: AIC, BIC and Laird's criterion (Laird, 1978). Each column in each block sums to 1000 (the total number of simulations per case). The green cells highlight the total number of simulations in which the best model estimates are equal to right values ($K = 1$ in the first column, $K = 2$ in the second column, $K = 3$ in the third column and $K = 4$ in the fourth column).

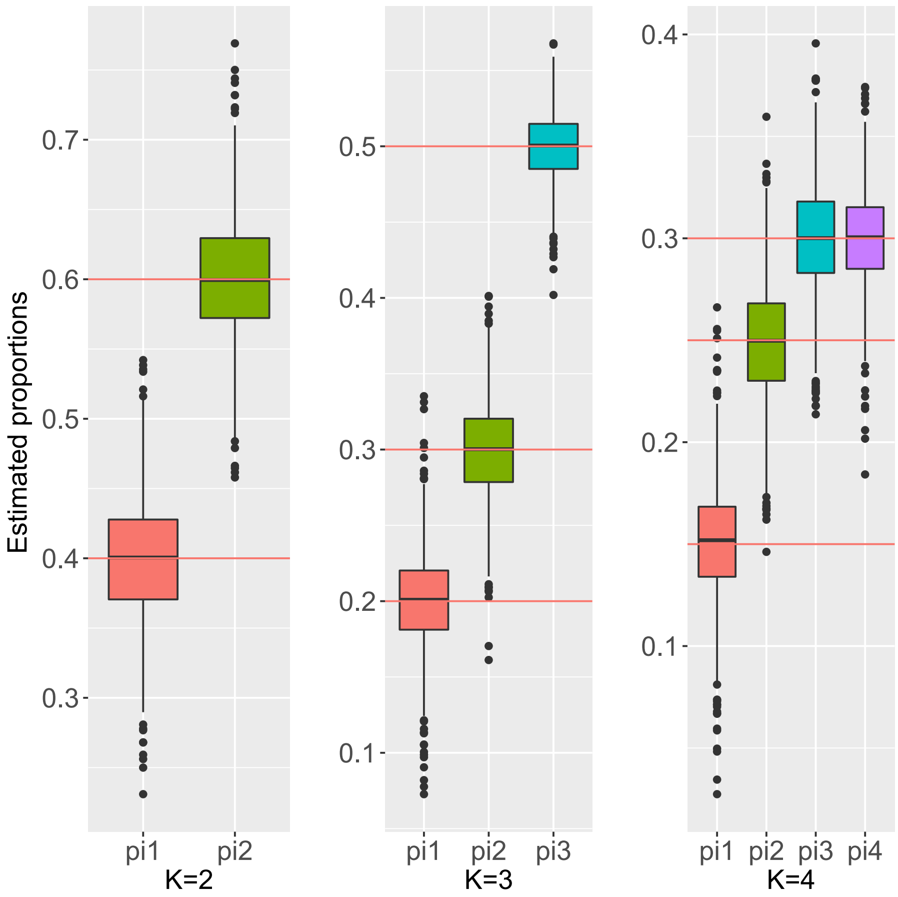

Fig. E.1. Estimates of the mixing proportions. In the first boxplot from the left we have $K = 2$ and the real values are $\boldsymbol{w} = [0.4, 0.6]$, in the second we have $K = 3$ and the real values are $\boldsymbol{\pi} = [0.2, 0.3, 0.5]$, in the third we have $K = 4$ and the real values are $\boldsymbol{\pi} = [0.15, 0.25, 0.30, 0.30]$. The red lines represent the real values.

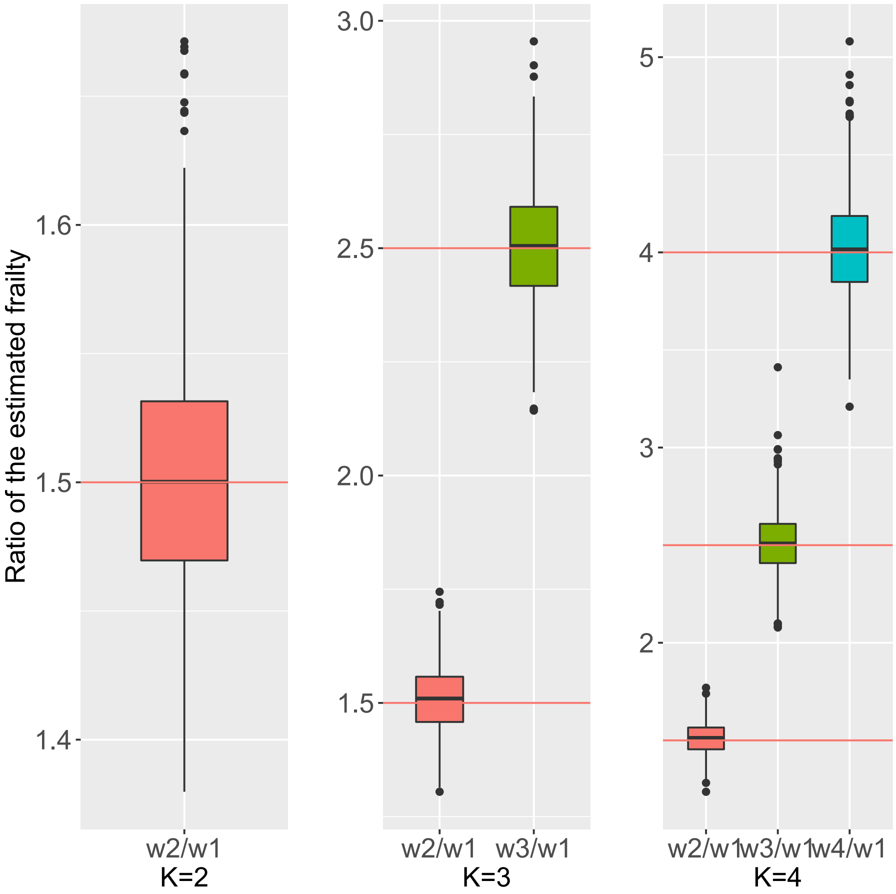

Fig. E.2. Ratios of the estimated frailties. In the first boxplot from the left we have $K = 2$ and the real values are $w_2/w_1 = 1.5$, in the second we have $K = 3$ and the real values are $\mathbf{w}/w_1 = [1.5, 2.5]$, in the third we have $K = 4$ and the real values are $\mathbf{w}/w_1 = [1.5, 2.5, 4]$. The red lines represent the real values.
